# Biotechnological Approaches for Host Plant Resistance to Insect Pests

**DOI:** 10.3389/fgene.2022.914029

**Published:** 2022-06-02

**Authors:** Pritam Kumari, Poonam Jasrotia, Deepak Kumar, Prem Lal Kashyap, Satish Kumar, Chandra Nath Mishra, Sudheer Kumar, Gyanendra Pratap Singh

**Affiliations:** ^1^ ICAR-Indian Institute of Wheat and Barley Research, Karnal, India; ^2^ CCS Haryana Agricultural University, Hisar, India

**Keywords:** biotic stress, insect pests, insecticide, biotechnology, resistant variety

## Abstract

Annually, the cost of insect pest control in agriculture crosses billions of dollars around the world. Until recently, broad-spectrum synthetic pesticides were considered as the most effective means of pest control in agriculture. However, over the years, the overreliance on pesticides has caused adverse effects on beneficial insects, human health and the environment, and has led to the development of pesticide resistant insects. There is a critical need for the development of alternative pest management strategies aiming for minimum use of pesticides and conservation of natural enemies for maintaining the ecological balance of the environment. Host plant resistance plays a vital role in integrated pest management but the development of insect-resistant varieties through conventional ways of host plant resistance takes time, and is challenging as it involves many quantitative traits positioned at various loci. Biotechnological approaches such as gene editing, gene transformation, marker-assisted selection etc. in this direction have recently opened up a new era of insect control options. These could contribute towards about exploring a much wider array of novel insecticidal genes that would otherwise be beyond the scope of conventional breeding. Biotechnological interventions can alter the gene expression level and pattern as well as the development of transgenic varieties with insecticidal genes and can improve pest management by providing access to novel molecules. This review will discuss the emerging biotechnological tools available to develop insect-resistant engineered crop genotypes with a better ability to resist the attack of insect pests.

## 1 Introduction

Under the changing climate scenario, the world’s population is estimated to increase by 2 billion in the next 30 years, rising from the current 7.7 billion population to 10 billion by 2050 (Zsögön et al., 2022). In this context, there is a continuous need for an increment of food production to fulfill the need of the rising worldwide population. Additionally, it is assessed that total global food demand will increase from 35% in 2010 to 56% in 2050 (van Dijk et al., 2021). To meet these goals it is critical to increase crop yield and reduce pre- and post-harvest losses. Also despite of operating control measures, a large portion of the economically significant harvests experiences a wide range of yield losses. Population extension, depletion of natural resources, environmental change, and developing insect pests are the key limitations that contrarily affect overall agricultural production and productivity (Alemu, 2020). Amongst these production constraints, biotic factors address one of the foremost constraints to crop productivity. Amongst the biotic factors, the insect pests are assessed to cause 25%–30% losses to agricultural production (Joshi et al., 2020; Mateos Fernandez et al., 2021). This suggests that insect pests pose a severe danger to food security and sustainable development, necessitating the development of effective plant protection technologies to prevent and control pest-related crop losses (Oerke et al., 2006). Chemical pesticides provide the first line of defense for farmers against insect pests, and their widespread use has resulted in a number of issues, including the killing of the beneficial insects, environmental pollution (Pedigo and Rice, 2006; Stevens et al., 2012), human and animal health problems and; imparting resistance in pests (Nderitu et al., 2020). To meet these challenges, it is necessary to move towards more sustainable and modern agricultural practices. Moreover, these detrimental non-target effects have motivated researchers around the world to develop novel and environment-friendly, alternative insect pest management strategies. Therefore, host plant resistance can form the backbone of pest management in different agro-ecosystems (Sharma, 2007).

**FIGURE 1:**
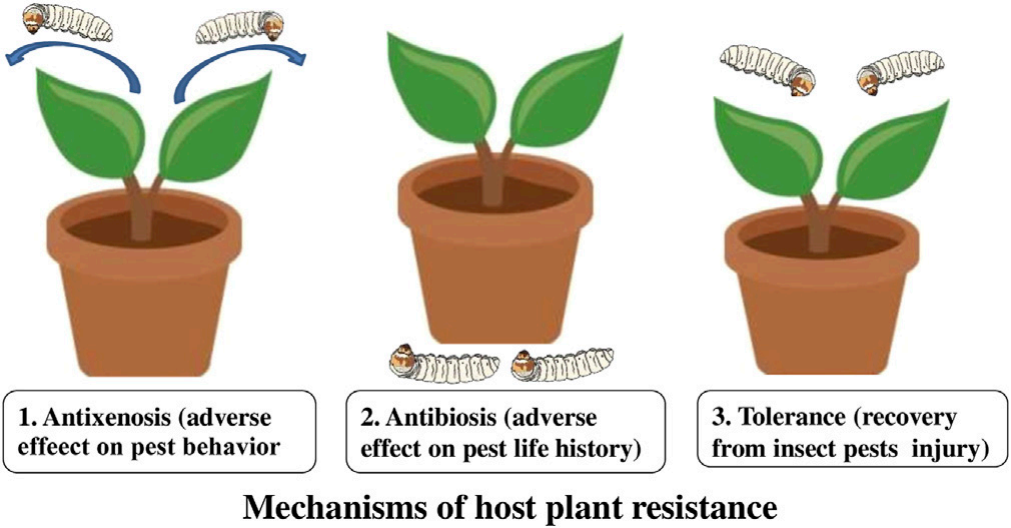
Mechanism of host plant resistance in insects.

Host plant resistance is the key component of pest management and one of the most appreciated control tactics in advanced agriculture (Horgan et al., 2020; El-Dessouki et al., 2022). It is the consequences of heritable plant characteristics that make a plant to be less damaged than a plant lacking these qualities. Insect-resistant crop varieties reduce the number of insect pests by increasing their tolerance for injury. Three types of resistance determine the relationship between the insect and the plant, e.g. antibiosis, antixenosis (non-preference), and tolerance (Koch et al., 2016; Iqbal et al., 2018) (Figure 1). Antibiosis resistance influences the biology of the pest to diminish its population and subsequent damage, resulting in higher mortality or reduced longevity and reproduction of the insect. Antixenosis resistance is defined as non-preference of the pest for a resistant plant and influences the behavioral traits of a pest (Painter, 1951; Smith, 2005). Tolerance is a resistance where a plant can resist or recover from damage caused by the pest population (Smith, 2005).

The development of insect-resistant plants began in 1782 when Havens published an article on a Hessian fly-resistant wheat cultivar. Since that time, several insect-resistant cultivars have been developed by the international and national research centers, the private sector using conventional or biotechnological tools (Jaiswal et al., 2018). The major biochemical principles underlying such resistance and the genes included have been distinguished for their coordinated utilization through biotechnological advancements in the course of the most recent 30 years (Joshi et al., 2020). Furthermore, for global food security and agricultural sustainability, contemporary agriculture’s primary goal is to enhance yields using existing land and resources. Therefore, innovative technologies have to be exploited to control pests and ensure adequate food availability in the future. Different plant protection innovations have been created to control, prevent and manage these pests with the trend of emphasizing/concentrating on the use of newer and more advance biotechnological approaches that are proven to be most efficient and provide results in a very short time as compared to conventional methods. These approaches can act as the backbone of crop protection against a broad spectrum of insect pests. In this due consideration, modern biotechnology put forward the best possible alternatives for diversifying agricultural production by accelerating the development of new insect resistant varieties of cereals, horticultural and even underutilized crops (Abah et al., 2010). Therefore, the main objective of this review is to assess the opportunities provided by these new biotechnological tools in developing various crop plants that are resistant to a wide range of insect pests.

## 2 Biotechnological Approaches in Insect Pest Management

Biotechnology can be broadly defined as “a method of creating or modifying a product, improving plants or animals, or developing microorganisms for specific purposes by utilising biological systems, living organisms, or derivatives thereof” (Persley, 2000). However, it can be described as the regulated and deliberate manipulation of biological processes to accomplish efficient insect pest control. From insect resistance breeding to transgenic introgression of novel genes, biotechnological interventions in insect pest management to protect crop yield have been enormous. The different biotechnological approaches include gene transformation, genome editing, RNA interference, marker-assisted selection, anther culture, embryo culture, protoplast fusion, somaclonal variations etc. (Talakayala et al., 2020). These approaches are described in detail below.

### 2.1 Gene Transformation

Gene transformation or genetic engineering of crops for insect resistance involves incorporation of specific DNA segment or gene into crop plants to provide resistance against insect pests. The DNA segment which get introduced usually encode a protein with insecticidal activity. Resistance is plant is conferred against specific insect pest through the expression of an insecticidal protein present in the introduced DNA segment (Gatehouse, 2013). This technology has been tested against a wide range of insect pests belonging to orders; Lepidoptera, Coleoptera, and Diptera (Birkett and Pickett, 2014). Genetically modified crops producing insecticidal proteins from *Bacillus thuringiensis*, a soil bacterium, have been widely used in agriculture globally since their introduction in 1996 (Abbas, 2018). The *cry* gene transformation technology involves the transfer of specified DNA sequences or genes into crop plants via *Agrobacterium*-mediated transformation or particle bombardment (Juturu et al., 2015). Apart from this, other strategies to protect plant from insects attack have also been investigated. Lectins, usually found in a number of plants, bind to carbohydrates in the midguts of phytophagous insects disrupting the digestive system (Vandenborre et al., 2011). Transgenic techniques have also been used to deploy protease inhibitors, which are designed to prevent insects from digesting their food (Singh et al., 2020). Similarly, transgenic plants expressing alpha-amylase inhibitors have been produced which are resistant to Lepidopterans, Coleoptrans, Dipterans and Hemipterans insects. Also, insect chitinase and chitinase-like proteins play a significant role in degrading chitin in the exoskeletal and gut linings of insects. These have been successfully cloned into plants and show insecticidal properties. Each of these strategies along with their role in insect pest management is discussed in detail below:

#### 2.1.1 Cry Genes

**FIGURE 2:**
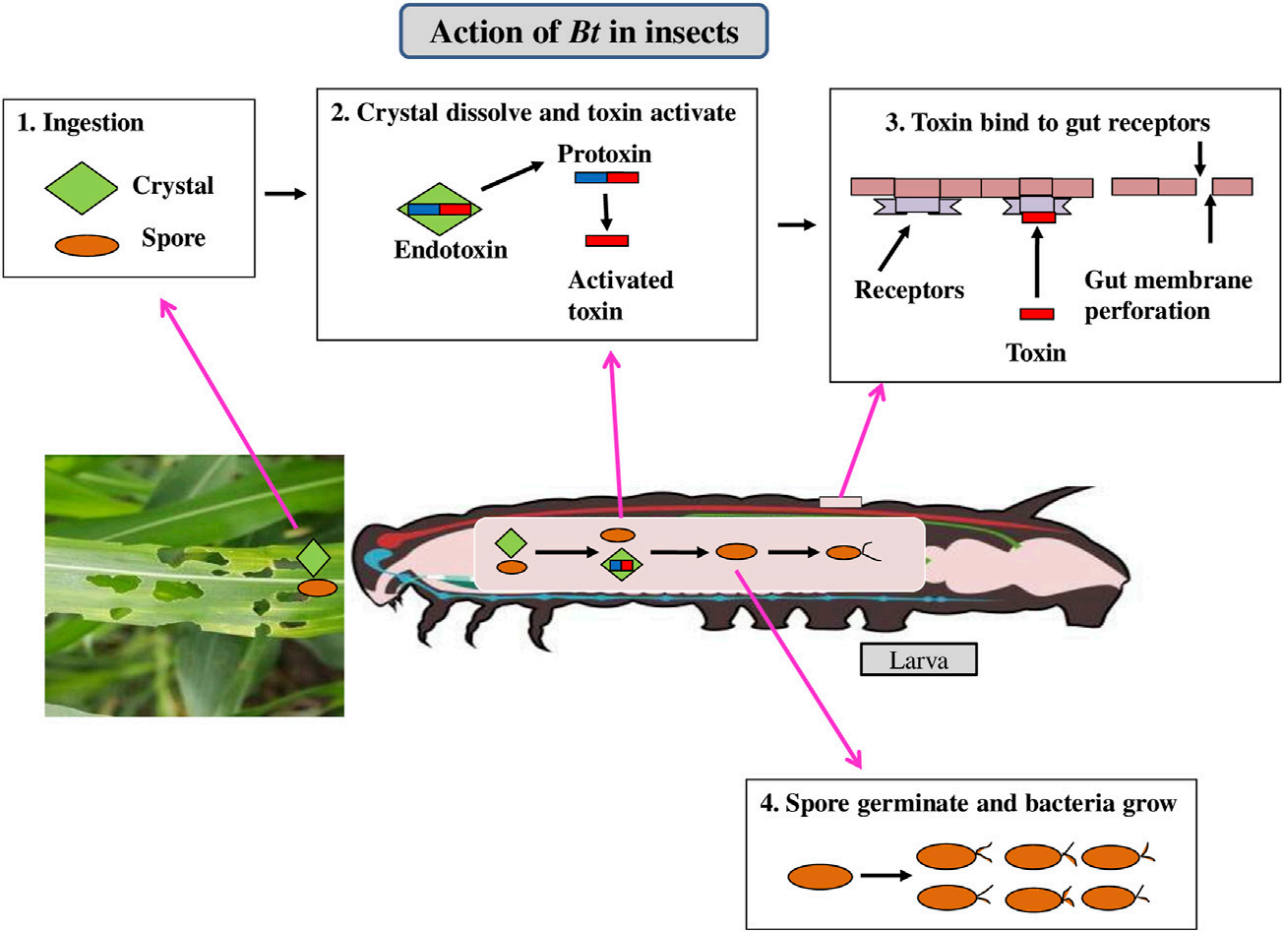
Mechanism of action of *Bt* cry toxin in insects.

*Bacillus thuringiensis* (*Bt*) is a Gram-positive soil bacterium expressing insecticidal crystalline proteins (ICPs) that are exceptionally toxic to specific classes of pests (Panwar et al., 2018). Insecticidal activity in insect-resistant *Bt* crops is expressed by the genes coding for parasporal crystal protoxins (Palma et al., 2014). ICPs produced by transgenic plants have had a significant impact on the successful evolution of insect resistance. The crystal involves a protoxin protein which get solubilized in the larval midgut due to alkaline pH and subsequently cleaved enzymatically to an active toxin. The toxin diffuses through the peritrophic membrane covering the gut and binds to receptors present in the midgut epithelium (Paul and Das, 2020) making pores in the midgut epithelium. The gut gets paralyzed and then the pest stops feeding and dies within 2–3 days (Figure 2).

The first-generation *Bt* cotton, Bollgard I (BG I) expressing*cry1Ac* was commercialized and released in 2002 to control the dominant bollworms including *Pectinophora gossypiella, Earias vittella,* and *Helicoverpa armigera* in cotton-cultivating areas of India. After thatBollgard II (BG II) was launched in 2006 as a second-generation *Bt* cotton with pyramided traits expressing *cry1Ac* and *cry2Ab* (MON15985 event), which is now cultivated in 95% of the total India’s cotton sowing area. In comparison to BG I containing *cry 1Ac* alone, BG II having multiple toxins, *cry1Ac* and *cry2Ab* have greater ability for pest management (Carrière et al., 2015). Another transgenic cotton *i.e.,* wide strike cotton expressing *cry1Ac* + *cry1F* was approved in the USA in the year 2004 by Dow Agro Sciences which improved both the crop yield as well as farmer’s income. It is observed that both BG II and wide-strike cotton expressing pyramided traits have a higher potential to suppress a wide range of Lepidopteran, Coleopteran and Dipteran insects than BG I. Another transgenic cotton containing *cry10Aa* exhibited strong resistance to cotton boll weevil (*Anthonomous grandis*) with 100% mortality observed through bioassay testing when the larvae of T_1_ generation consumed the leaves of transgenic cotton (Ribeiro et al., 2017). *Agrobacterium*-mediated transformation was employed to produce transgenic cotton expressing pyramided traits, *cry1Ac* and *cry2Ab* cloned in the T-DNA, and the resulting plant demonstrated resistance to *S. litura* with 93 percent larval mortality (Siddiqui et al., 2019). Transgenic rice lines constructed through the expression of the *cry2AX1* gene showed resistance to rice leaf folder (*C. medinalis*) and rice yellow stem borer (*S. incertulas*) (Rajadurai et al., 2018).

Transgenic cotton and brinjal cultivars having resistance to borers were permitted for commercial usage in Bangladesh and in Latin America, insect-resistant Bt soybean expressing cry1Ac + cry1Ab were allowed for production during 2014 (Koch et al., 2015). Another study found that a synthetic *cry1Ab* gene introduced into tomato conferred resistance to the tomato leaf miner, *T. absoluta*, with 100 percent insect mortality at T_0_ generation within 4–5 days (Soliman et al., 2021). Rice lines (var. Bg94–1) produced by transferring the insecticidal protein *cry2A* cause mortality in rice leaf folders in 80% of cases (Gunasekara et al., 2017). Similarly, transgenic pigeon pea lines constructed using a combination of *cry1Ac* and *cry2Aa* exhibited resistance to *H. armigera*, resulting in 80%–100% larval mortality (Ghosh et al., 2017). *Cry1Aa* gene expression in sweet potatoes conferred resistance to Lepidopteran insect *i. e, Spodoptera litura* (Zhong et al., 2019). Expression of *cry2AX* gene in transgenic cotton event CH12 showed 88% mortality in *H. armigera* at T_0_ generation (Sakthi et al., 2015). et alAn industrially important non-edible castor developed by transferring the *cry1Aa gene* using *Agrobacterium* transformation technique, exhibited strong resistance against two lepidopteran pests, *i.e*., *Achaea janata* (semi-looper) and *S. litura* (Muddanuru et al., 2019). Transgenic soybean, expressing *cry8*-like gene from *B. thuringenesis* conferred resistance to *Holotrichia parallela*, a Coleopteran pest (Qin et al., 2019). The transgenic cotton event MNH93 carrying *cry1Ab* demonstrated 40–60 percent larval mortality against *H. armigera* with displaying 0.26 percent transformation frequency (Khan et al., 2011). In *H. armigera*, expression of the *cry2AXI* gene in the T_3_ generation of the cotton event CH12 resulted in 90% death (Jadhav et al., 2020). Other toxins or proteins, such as *cyt2Aa*, which provides aphid resistance (Chougule et al., 2013) and *cry51Aa2*, increased *Lygus* species mortality in cotton (Gowda et al., 2016). The expression of *cry* gene has been studied in different crop species and is depicted in Table 1. Despite the successful deployment of cry gene technologies in crops to achieve resistance against several insect pests, some of the agricultural pests often develop resistance to insecticidal toxins and devastate the crop production. Other problems that limit the usefulness of transgenic crops for insect control include secondary pest outbreak, evolution of new biotypes, effects on non-target organisms, environmental influences on gene expression, biosafety of food from transgenic crops, and socio-economic and ethical issues. Also the research groups should take up the challenges of understanding plant insect interactions to understand the mechanism of resistance development in insects against *cry* genes.

**TABLE 1:** Transgenic crops carrying *Bt* genes for insect resistance.

S. No.	Target insects	Transgene	Target crop	References
1	*Chilo suppressalis*, *Cnaphalocrocis medinalis*	*cry 1a(b)*	Rice	Fujimoto et al. (1993)
2	*Scirpophaga incertulas* & *Chilo suppressalis*	*cry 1 a(b)*	Rice	Wünn et al. (1996)
3	*Scirpophaga incertulas*, *Cnaphalocrocis medinalis*	*cry 1 a(b)*	Rice	Ghareyazie et al. (1997)
4	*Scirpophaga incertulas*	*cry 1a(c)*	Rice	Nayak et al. (1997)
5	*Scirpophaga incertulas*	*cry 1a(b)/cry1a(c)*	Rice	Tu et al. (2000)
6	*Cnaphalocrocis medinalis*, *Scirpophaga incertulas*	*cry 2a/cry 1a(c)*	Rice	Maqbool et al. (2001)
7	*Helicoverpa armigera*	*cry1Ab + NptII*	Cotton	Khan et al. (2011)
8	*Heliothis* sp.	*cry1Ab*	Cotton	Khan et al. (2013)
9	*Helicoverpa armigera*	*cry2AX*	Cotton	Sakthi et al. (2015)
10	*Helicoverpa armigera*	*cry1AC + cry2Aa*	Pigeon pea	Ghosh et al. (2017)
11	*Helicoverpa armigera*	*cryIIAa*	Chickpea	Sawardekar et al. (2017)
12	*Cnephalocrosis medinalis*	*cry2A*	Rice	Gunasekara et al. (2017)
13	*Tuta absoluta*	*cry1Ac*	Tomato	Selale et al. (2017)
14	*Anthamous grandis*	*cry1Aa*	Cotton	Ribeiro et al. (2017)
15	*Helicoverpa armigera*	*cry2Aa*	Pigeon pea	Baburao and Sumangala (2018)
16	*Helicoverpa armigera*	*cry2Aa*	Pigeon pea	Singh et al. (2018)
17	*Scirpophaga incertulas*, *Cnaphalocrocis medinalis*	*cry2AX1*	Rice	Rajadurai et al. (2018)
18	*Spodoptera litura*	*cry1Aa*	Sweet Potato	Zhong et al. (2019)
19	*Spodoptera litura*	*cry1AC + cry2Ab*	Cotton	Siddiqui et al. (2019)
20	*Holtrichia panallele*	*cry 8 like*	Soyabean	Qin et al. (2019)
21	*Achaea janata, Spodoptera litura*	*cry1AC*	Castor	Muddanuru et al. (2019)
22	*Helicoverpa armigera*	*cry2AX1*	Cotton	Jadhav et al. (2020)
23	*Tuta absoluta*	*cry1Ab*	Tomato	Soliman et al. (2021)

#### 2.1.2 Vegetative Insecticidal Proteins Genes

The *Bacillus thuringiensis* (*Bt*) bacteria found in a variety of ecological habitats, has natural entomo-pesticidal properties against a variety of economically important crop pests due to the secretion of various proteins during different growth phases. One of the best known families of *Bt* proteins is Vip, which are produced during the vegetative growth phase of the plant and are recognized as an excellent toxic candidate due to the sequence homology and receptor sites differences from *cry* proteins. There are three subfamilies of Vip proteins. Vip1 and Vip2 heterodimer toxins, effective against pests belonging to Hemiptera and Coleoptera orders, whereas Vip3, the most extensively studied family of Vip toxins have toxicity toward Lepidopterans et al. Vip proteins are also known as second-generation insecticidal proteins, that can be used either alone or in combination with *cry* proteins to control a number of insect-pests (Gupta et al., 2021). In terms of toxicity potential against susceptible insects, these Vip3 proteins are comparable to *cry* proteins. They are reported to be toxic toward pests, which can’t be controlled with *cry* proteins. They reduce the insect pest’s population by osmotic lysis which causes swelling and interruption of the midgut epithelial cells. The Vip3 proteins have been successfully pyramided along with *cry* proteins in transgenic crops such as maize and cotton, to overcome resistant pest populations and delay the evolution of resistance (Syed et al., 2020).

Vip genes exhibiting greater resistance against cotton bollworm (*H. armigera*) and tobacco budworm (*Heliothis virescens*) showing that these genes are an excellent option for these insect control. The transgenic cotton containing Vip3A alone and another multitoxin line expressing Vip3A and *cry1Ab* (VipCot) had greater resistance to both the insects, *H. zea* and *H. virescens* (Bommireddy et al., 2011). Transgenic cotton lines expressing Vip3AcAaa demonstrated greater insect resistance, showing that the Vip3AcAaa protein is highly effective in insect control (Chen et al., 2018a). The cowpea lines containing Vip3Ba1 exhibited greater suppression of larval growth and showed further resistance against the pod borer (Bett et al., 2017). A toxin Vip3A transferred in sugarcane showed superior resistance against sugarcane stem borer (*Chilo infuscatellus*) with 100% mortality (Riaz et al., 2020). The effect of the Vip gene in different crop species is depicted in Table 2. These proteins are promising candidates for further development of insect resistant plants and show extended ranges of toxicity particularly toward lepidopteran pests. Efforts are underway to use these proteins to induce insect pest resistance.

**TABLE 2:** Expression of *VIP* genes for insect resistance.

S. No.	Target insects	Transgene	Target crop	References
1	*Heliothis. zea* and *H. virescens*	*Vip3A + cry1Ab*	Cotton	Bommireddy et al. (2011)
2	*Maruca vitrata*	*Vip3Ba1*	Cowpea	Bett et al. (2017)
3	*Helicoverpa armigera*	*Vip3AcAaa (Vip3Aa1+Vip3Ac1)*	Cotton	Chen et al. (2018a)
4	*Chilo infuscatellus*	*Vip3A*	Sugarcane	Riaz et al. (2020)

#### 2.1.3 Lectins

Carbohydrate-binding proteins known as lectins, are entomotoxic proteins with insecticidal properties and are found in many plant species. They prevent predation by being detrimental to a variety of insects and animals that eat plants. They are mostly found in plants belonging to the families Solanaceae, Fabaceae and Poaceae; especially some of leguminous seeds contain a high concentration of lectins. Plant lectins act as storage proteins and are involved in defense mechanisms against phytophagus insects. Various plants lectins from different sources have already been reported to be toxic towards important members of insects belonging to Lepidoptera (Czapla and Lang, 1990), Coleoptera (Gatehouse et al., 1984; Czapla and Lang, 1990) and Homoptera orders (Powell et al., 1993; Sauvion et al., 1996). The first lectin discovered and commercially available was Concanavalin A; which is now the most extensively studied lectin for insect pest control. The adverse impact of lectins on biological parameters of insects includes, feeding inhibition, reduction in larval weight delays in adult emergence, retardation in total developmental period and increased mortality and reduced fecundity in the first and second generation (Powell et al., 1993).

Insect-resistant plants have emerged in recent years, paving the way for the use of plant lectins in pest management strategies. The use of lectins in transgenic plants has yielded promising results, particularly for crops expressing *Bacillus thuringiensis* (*Bt*) Cry toxins, which show resistance to sap-sucking insects. Furthermore, lectins in artificial diets and their expression in transgenic plants have been shown to reduce performance in insects of various orders, including Lepidoptera, Coleoptera, and Hemiptera.

Plant lectins are carbohydrate-binding proteins that have greater affinity for certain sugar components found in glycoproteins and glycolipids in the cell membrane (Camaroti et al., 2017). Transgenic rice carrying *Allium sativum* leaf agglutinin (ASAL) and *Galanthus nivalis* lectin (GNA) conferred resistance to major sap-sucking insects like brown planthoppers (BPH), white-backed planthoppers (WBPH), and green leafhoppers (GLH) (Bharathi et al., 2011). Transgenic rice (*Oryza sativa* L.) plants expressing an insecticidal protein (the snowdrop lectin, GNA) produced through particle bombardment exhibited the significant levels of resistance against sap-sucking pests (Sudhakar et al., 1998). Enhanced toxicity of GNA-spider-venom toxin I (SFI1) fusion protein to larvae of the tomato moth (*L. oleracea*), rice brown planthopper (*N. lugens*), and the peach potato aphid (*M. persicae*) reported by Fitches et al. (2004) and Down et al. (2006). In an another study, the harmful effects of snowdrop lectin (GNA) expressed in transgenic sugarcane on the life cycle of Mexican rice borer *Eoreuma loftini* (Dyar) and sugarcane borer *Diatraea saccharalis* (F.) was reported by Sétamou et al. (2002). Expression of *Galanthus nivalis* lectin (GNA) gene showed resistance to aphids in potato (Mi et al., 2017). Transgenic *Arabidopsis* expressing recombinant fusion protein (Hv1a/GNA) exhibited resistance to peach potato aphids and grain aphids (Nakasu et al., 2014). A GNA-neuropeptide-allatostatin fusion protein was found to inhibit the feeding and growth of the tomato moth (*L. oleracea*). (Fitches et al., 2004). The larvae of the tomato moth were found to be more toxic to a fusion protein containing a GNA-lepidopteran-specific toxin (ButalT) from the South Indian red scorpion (*Mesobuthus tamulus*). (Trung et al., 2006). The transgenic cotton lines containing insect gut binding lectin demonstrated significant level of resistance to sucking and chewing insects at T_1_ generation (Vanti et al., 2018). The lentil lectin (LL) and chickpea protease inhibitor (CPPI) genes transferred to *Brassica juncea* lines and the resulting transgenic plants showed resistance to sap-sucking pests such as aphids (Rani et al., 2017b). Mannose-binding lectin expressed through *Agrobacterium*-mediated transformation, exhibited resistance to wheat aphid in BE104 (Duan et al., 2018). In a bioassay study it was found that the transgenic wheat plants produced through the particle bombardment method expressing the gene encoding snowdrop lectin (*Galanthus nivalis* agglutinin; GNA) showed reduced fecundity of grain aphids *Sitobion avenae* (Stoger et al., 1999). At T_1_ and T_2_ generations, transgenic *B. juncea* expressed a new lectin gene, *Colocasia esculenta* tuber agglutinin (CEA), as well as a GNA, exhibited enhanced resistance against mustard aphid (*Lipaphis erysimi*) (Das et al., 2018). Transgenic cowpea expressing *arcelin 1* gene from *Phaseolus vulgaris* L. conferred greater resistance to bruchids like *Zabrotes subfasciatus* and *Callosobruchus maculatus*. In addition, against both bruchid insects, the percentage of eggs laid, hatching, adult emergence, and grain mass loss was much lower in transgenic cowpea than in control (Grazziotin et al., 2020). Hilda et al. (2022) demonstrated that arcelin found in the wild accession of common bean is an insecticidal protein that can prevent the bruchid beetle (Bruchidae: Coleoptera) from digesting it. Aside from focusing on coleopteran insects, arcelin was reported to be highly effective against specific insects that belong to Lepidoptera and Hemiptera insect order (Oriani and Lara, 2000; Malaikozhundan et al., 2003). The binding of lectin molecules to glycosylated proteins in the midgut of larvae reduces the efficiency of nutrient uptake and diet utilisation, resulting in a drop in total larval mass and decline in the average survival rate. (Paiva et al., 2012). Caroline et al. (2022) reported that Erythroagglutinin, Arcelin-5, Leucoagglutinin, and a hypothetical seed storage protein are responsible for bruchid resistance which is among the most devastating insect pest of the common bean. Findings of Khoobdel et al. (2022) indicated that a lectin derived from *Polygonum persicaria* L. (PPA), causes oxidative stress in *Sitophilus oryzae* in addition to causing digestive disorders,. The leaf lectin (SteLL) from *Schinus terebinthifolius* did not cause death in *S. zeamais* adults, according to Camaroti et al. (2018) and de Santana Souza et al. (2018), but it did inhibit protease activity and promote amylase activity in the digestive system. MvRL was found to affect the activity of gut endoglucanase, phosphatases, b-glucosidase, and trypsin-like enzymes in *Nasutitermes corniger* workers and soldiers, reported by de Albuquerque et al. (2012).

Plant lectins have been found to be biologically active against a variety of insects. Chitin-binding lectins derived from *Microgramma vacciniifolia* rhizome lectin (MvRL) showed anti-nutritional effects on survival, feeding, and nutrition of *Sitophilus zeamais* adults (de Albuquerque et al., 2020). *Moringa oleifera* seeds containing a water-soluble lectin (WSMoL) negatively impacted the physiology of the pest *Sitophilus zeamais*, which could have long-term consequences (de Oliveira et al., 2020). According to Napoleão et al. (2013), the leaf lectin (MuLL) derived from *M. urundeuva* adversely affected the activity of digestive enzymes in the stomachs of *S. zeamais* adults, inhibiting digestive processes. Seeds containing the lectin WSMoL (water-soluble *M. oleifera* lectin) have been shown to be insecticidal against *Aedes aegypti* eggs and larvae. (Coelho et al., 2009; Santos et al., 2012, Santos et al., 2020). The survival and nutritional parameters of *S. zeamais* adults are negatively affected by the ingestion of an artificial diet containing a saline concentrate from *Schinus terebinthifolius* Raddi leaves (LE) or its lectin (SteLL, *S. terebinthifolius* leaf lectin). (Camaroti et al., 2018). However, progress in lectin research has been hampered due to concerns about the effects of ingesting snowdrop lectin on higher animals,, although no adverse effect was seen in rats fed for 90 days on transgenic rice containing GNA was seen (Poulsen et al., 2007). Insect resistance was demonstrated by the expression of lectin genes in various crop plants (Table 3). Although, lectins have been found to have negative effects on insect pests of various orders and stages of development, preventing growth, survival, nutrition, development, and reproduction (Napoleão et al., 2019). However, because of their known toxicity to mammals and humans, caution should be exercised in their use in transgenic plants.

**TABLE 3:** Expression of lectin genes for insect resistance.

Sr. No.	Target insects	Transgene	Target crop	References
1	Sap sucking pests	Snowdrop lectin (*Galanthus nivalis* agglutinin; GNA)	Rice	Sudhakar et al. (1998)
2	*Sitobion avenae*	Snowdrop lectin (*Galanthus nivalis* agglutinin; GNA)	Wheat	Stoger et al. (1999)
3	*Eoreuma loftini* (Dyar) and *Diatraea saccharalis*	Snowdrop lectin (*Galanthus nivalis* agglutinin; GNA)	Rice, Sugarcane	Sétamou et al. (2002)
4	*Lacanobia oleracea*	GNA-neuropeptide-allatostatin	Tomato	Fitches et al. (2004)
5	*Lacanobia oleracea*	GNA-lepidopteran-specific toxin (ButalT)	Tomato	Trung et al. (2006)
6	*Nilaparvata lugens and Myzus persicae*)	GNA-spider-venom toxin I (SFI1)	Rice and Potato	Down et al. (2006)
7	*Aedes aegypti* eggs and larvae	WSMoL (water-soluble *M. oleifera* lectin)	-	Coelho et al. (2009); Santos et al. (2012), Santos et al. (2020)
8	*Nilaparvata lugens, Sogatella furcifera and Nephotettix nigropictus*	*Allium sativum* leaf agglutinin (ASAL) and *Galanthus nivalis* lectin (GNA)	Rice	Bharathi et al. (2011)
9	*Nasutitermes corniger* workers and soldiers	Endoglucanase, phosphatases, b-glucosidase, and trypsin	-	de Albuquerque et al. (2012)
10	*Sitophilus zeamais*	*M. urundeuva* leaf lectin (MuLL)	Stored grains	Napoleão et al. (2013)
11	*Myzus persicae* and *Sitobion avenae*	Hv1a/GNA	Potato	Nakasu et al. (2014)
12	*Myzus persicae*	*Galanthus nivalis* agglutinin (GNA)	Potato	Mi et al. (2017)
13	*Lipaphis erysimi*	Lentil lectin (LL) and Chickpea protease inhibitor (CPPI) genes	Transgenic *Brassica juncea*	Rani et al. (2017b)
14	*Sitophilus zeamais*	*Schinus terebinthifolius* leaf lectin (SteLL)	Stored grains	de Santana Souza et al. (2018)
15	*Lipaphis erysim*i	*Colocasia esculenta* tuber agglutinin (CEA)+ *Galanthus nivalis* agglutinin (GNA)	Mustard	Das et al. (2018)
16	*Metopolophium dirhodum, Schizaphis graminum, Rhopalosiphum padi,* and *Sitobion avenae*	*Pinellia pedatisecta* agglutinin (PPA)	Wheat	Duan et al. (2018)
17	*Aphis gossypii* and *Spodoptera litura*	Insect gut binding lectin from *Sclerotium rolfsii*	Cotton	Vanti et al. (2018)
18	*Callosobruchus chinensis*	Arcl on APA locus from *Phaeselous vulgaris*	Cowpea	Grazziotin et al. (2020)
19	*Sitophilus. zeamais*	*Microgramma vacciniifolia* rhizome lectin (MvRL)	Stored grains	de Albuquerque et al. (2020)
20	*Sitophilus zeamais*	Water-soluble *Moringa oleifera* lectin (WSMoL)	Stored grains	de Oliveira et al. (2020)
21	*Callosobruchus chinensis*	Arcelin	Common bean	Hilda et al. (2022)
22	*Callosobruchus chinensis*	Arcelin-5, Leucoagglutinin, Erythroagglutinin	common bean	Caroline et al. (2022)
23	*Sitophilus oryzae*	*Polygonum persicaria* L. (PPA) Lectin	Stored grains	Khoobdel et al (2022)

#### 2.1.4. Fusion Proteins

*Bt* insecticides are widely used, with *Bt* toxins accounting for up to 90% of microbiological insect control products. As a result, there’s a possibility that insects will become resistant to *Bt* toxins. (Tabashnik, 1994; Ferré et al., 1995). The diamondback moth (*Plutella xylostella*) has evolved resistance in some open field populations in response to repeated exposure to foliar sprays containing *Bt* proteins. (Perez and Shelton, 1997), whereas recessive mutant alleles can confer resistance to multiple *Bt* toxins in laboratory selection experiments with other insect pests (Tabashnik et al., 1996). The stacking or pyramiding of multiple transgenes in the same transgenic plant and the use of hybrid toxins against insect pests are two recent strategies to address potential limitations in conventional transgenic insect pest control. et alThus fusion proteins are formed by joining together different insecticidal proteins. In the host plant system after transcription and translation these proteins form a single polypeptide units which are more effective against phytophagous insects. Dow Agro Sciences’ transgenic cotton lines carrying a hybrid fusion protein, *cry1Be + cry1Fa*, demonstrated increased resistance to *S. litura* and *O. nubilalis* insects (Meade et al., 2017). Transgenic rice containing the *cry2AX1* (derivative of *cry2Aa* and *cry2Ac*) gene was viewed as resistant to lepidopteran insects, as indicated by Chakraborty et al. (2016). Koerniati et al. (2020) reported that the synthetic sugarcane expressing *cry1Ab + cry1Ac* fusion protein showed resistance against shoot borer. Agrobacterium-mediated transformation of transgenic *B. juncea* expressing a fusion protein derived from lectin and a protease inhibitor resulted in resistance to phytophagous aphids. (Rani et al., 2017a). In another case study, Chang et al. (2017) demonstrated that transgenic maize containing *cry1Ab/cry2Aj* fusion protein in kernel conferred higher mortality in *S. exigua*, a lepidopteran pest and *Harmonia axyridis*, a coleopteran pest. Transgenic rice line expressing *cry1Ac* + ASAL conferred durable and enhanced resistance to major insects such as yellow stem borer, leaf folder,, and brown plant hopper (Boddupally et al., 2018). Pyramiding of *cry1Ab* + vip3A showed resistance against the rice leaf folder and Asiatic rice borer (Xu et al., 2018). A significant level of protein expression was observed in transgenic rice lines carrying *cry2Aa + cry1Ca* protein lethal to Asiatic rice borer, *Chilo suppressalis* (Qiu et al., 2019). Katta et al. (2020) investigated that the transgenic plant developed by transferring a triple gene construct containing *cry2Ab + cry1F + cry1Ac* genes into an elite cotton variety (Narasimha) conferred a significant level of mortality to *H. armigera* and *S. litura* at T_2_ generation. An account of studies utilization of the fusion proteins used for insect resistance in crop plants for pest management is depicted in Table 4. Pyramiding of two or more genes is a sustainable strategy for achieving good management of lepidopteran, coleopteran and hemipteran insects pests. Furthermore, these innovations could pave the way for development of insect resistant crops by delaying the phenonmenon of resistance development in insects.

**TABLE 4:** Fusion proteins for insect resistance in crop plants.

Sr. No.	Target insects	Transgene	Target crop	References
1	*Scirpophaga incertulas*, *Cnaphalocrocis medinalis*	*cry2AX1 (cry2Aa + cry2Ac)*	Rice	Chakraborty et al. (2016)
2	*Lygus* spp.	*cry51Aa2*	Cotton	Gowda et al. (2016)
3	*Spodoptera exigua, Harmonia axyridis*	*cry1Ab/cry2Aj*	Maize	Chang et al. (2017)
4	*Spodoptera litura, Ostrinia nubialis*	*cry1Be + cry1Fa*	Cotton	Meade et al. (2017)
5	*Lipaphis erysimi*	Lentil lectin (LL) and chickpea protease inhibitor (CPPI) genes	*Brassica juncea*- mustard	Rani et al. (2017b)
6	*Scirpophaga incertulas*, *Cnaphalocrocis medinalis, Nilaparvata lugens*	*cry1AC + ASAL*	Rice	Boddupally et al. (2018)
7	*Ostrinia furnacalis, Cnaphalocrocis medinalis*	*cry1Ab + vip3A*	Rice	Xu et al. (2018)
8	*Chilo suppressalis*	*cry2Aa + cry1Ca*	Rice	Qiu et al. (2019)
9	*Helicoverpa armigera, Spodoptera litura*	*cry2Ab + cry1F + cry1AC*	Cotton	Katta et al. (2020)
10	*Scirphophaga excerptalis*	*cry2Aa + cry1Ca, cry1Ab + cry1Ac*	Sugarcane	Koerniati et al. (2020)

#### 2.1.5 Protease Inhibitors

**FIGURE 3:**
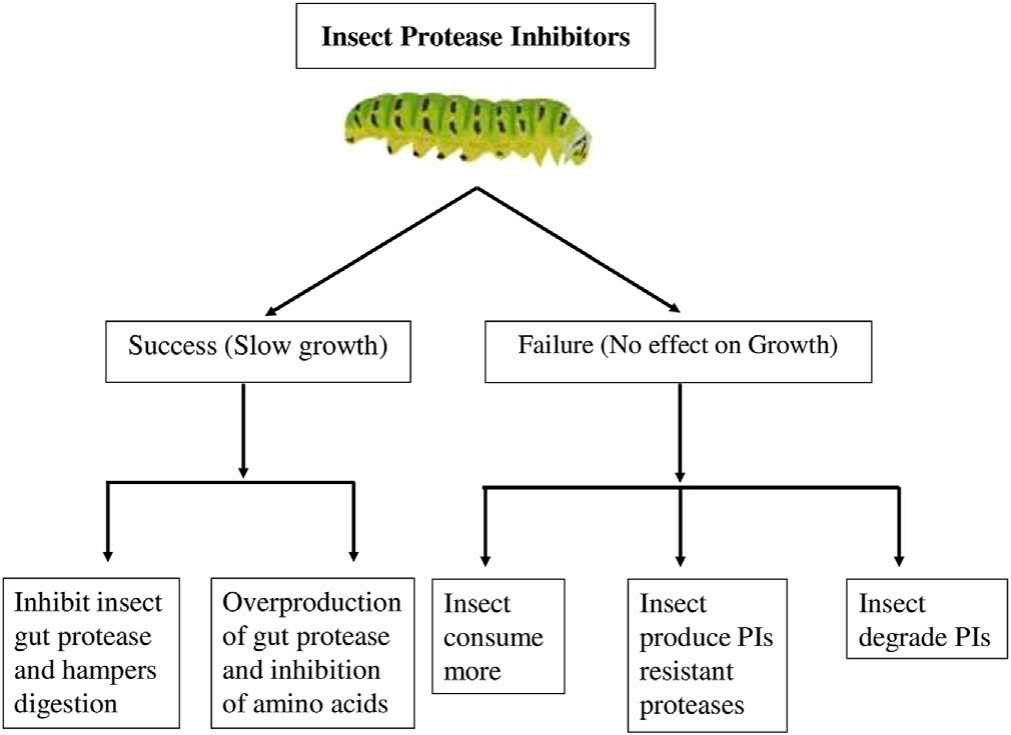
Impact of protease inhibitors (PIs) on insect pest growth.

Protease inhibitors (PIs) are plant-derived inhibitors that prevent insect pests from digesting their food by inhibiting the activity of digestive proteases (Haq et al., 2004; Macedo and Freire, 2011; Zhu-Salzman and Zeng, 2015). Insect digestive proteases are known to be inhibited by PIs, through preventing proteolysis and results in decreased fecundity, increased mortality and longer developmental period due to the deficiencies of essential amino acids. The most investigated plant PIs against pests are serpins and cystatins. Serpins, with a molecular mass of approximately 39–43 kDa, are irreversible serious inhibitors of serine proteases. Serine proteases have been discovered in insect orders like, Diptera (flies), Lepidoptera (moths and butterflies), Orthoptera (grasshoppers, locusts), Coleoptera (beetles) and Hymenoptera (bees and wasps) (Irving et al., 2002). Cystatins, a PIs protein with a molecular mass of 12–16 kDa, inhibit the activity of cysteine proteases, which are the primary digesting proteases in Coleopterans and Hemipterans. Several studies have also reported that volatile compounds such as methyl jasmonate, one of the key regulators of plants’ defensive response to insect herbivores, inhibit gut protease after wounding. (Singh et al., 2016), cause neighboring unwounded plants to produce proteinase inhibitors, effectively prearming the local population against insect attack (Stevens et al., 2012). Legume trypsin inhibitors inhibit a wide spectrum of proteases and have an insecticidal activity against a variety of key insects (Macedo et al., 2004; Sharma, 2015). Protease inhibitor genes were incorporated in rice cultivars (Duan et al., 1996; Xu et al., 1996) to improve protection against stem borers, and wheat (Altpeter et al., 1999) to protect them against foliage-feeding and storage pests. Protease inhibitors when fed to insect pests either through artificial diet or transgenic plants resulted into increased insect mortality (de Pg Gomes et al., 2005; Gatehouse, 2011) and adversely affected the growth and development of insect larvae from different insect orders (de Pg Gomes et al., 2005; A,; Gatehouse, 2011) (Burgess et al., 1994; De Leo et al., 2001; Outchkourov et al., 2004; Ribeiro et al., 2006; Tamhane et al., 2007; Dunse et al., 2010; Schneider et al., 2017). Figure 3 depicts the success and failure of protease inhibitors in a variety of insect pests. Protease inhibitors may enlighten a new dimension in insect pest management. However, due to lack of understanding of insect physiology and biochemistry, it suffered great failure in recent past. Also PIs turned worthless due to immense adaptive potential of insect pests and its long coevolutionary relationship with host plant. Solving these issues could pave the way for future research.

#### 2.1.6 α-Amylase Inhibitors

α-amylase is a digestive enzyme present in insects for digestion of carbohydrates. An α-amylase inhibitor, affect digestion in insects by inhibiting the activity of α-Amylase enzyme in insects. Various types of α-amylase inhibitors, present in seeds and vegetative organs of plants, was found to control a numbers of phytophagous insects (Chrispeels et al., 1998). Seeds of *Phaseolus vulgaris* expressing an α-amylase inhibitor negatively affected the growth and development of cowpea weevil *Callosobruchus maculatus* and Azuki bean weevil *Callosobruchus chinensis* (Ishimoto and Kitamura, 1989; Shade et al., 1994). Morton et al. (2000) reported that transgenic pea and azuki bean seeds expressing the inhibitor, aAI-1, exhibited enhanced resistance against bruchids, the pea weevil (*Bruchus pisorum*), the cowpea weevil (*Callosobruchus maculatus*) and the azuki bean weevil (*Callosobruchus chinensis*). Kaur et al. (2022) investigated that higher activity of α-amylase inhibitors in the central whorl leaves and stems of maize genotypes might be responsible for inducing resistance against *Chilo partellus* infestation. The multiplication and damage of *Rhyzopertha dominica*, a major pests of stored wheat grains can be effectively controlled by inhibiting the α-amylase enzyme through wheat α-amylase inhibitors (Priya et al., 2010). A gene named aAI-Pc1, encoding an α-amylase inhibitor was isolated from cotyledons of *Phaseolus coccineus* and introduced into coffee plants, confers resistance to coffee berry borer, *Hypothenemus hampei* (de Azevedo Pereira et al., 2006). Recently, a wheat gene encoding an α-amylase inhibitor was expressed in tobacco, resulting in increased resistance to *Spodaptera* spp. and *Agrotis* spp (Jaiswal et al., 2018). Isolation of a novel alpha-amylase inhibitor from papaya seeds (*Carica papaya*) showed increased larval mortality, decreased insect fecundity and adult longevity of cowpea weevil (*Callosobruchus maculates*) (Farias et al., 2007). Therefore these studies indicates the successful utilization of α-amylase inhibitors in insect pests management.

##### 2.1.6.1 Insect Chitinase

Insect chitinases are the hydrolytic enzymes having potential to inhibit or degrade the chitin. In insects, chitin is the main component of the exoskeleton and peritrophic membrane. It provide protection from harsh environmental conditions, external mechanical disruption and natural enemies (Chen et al., 2018b). The hydrolysis of chitin is essential for ecdysis (periodic shedding of the old cuticle). Chitinases are expressed in various organisms including those that lack chitin such as plants to recognize and degrade the chitin in chitin containing insects ((Oyeleye and Normi, 2018). Transgenic expression of chitinase enzyme has been proposed as a crop protection strategy. Role of chitinase enzyme in insect pests management has been studied in several insects such as silkworm *B. mori*, rice brown planthopper *N. lugens*, cotton mealybug *P. solenopsis and* rice striped stem borer *C. suppressalis* (Pan et al., 2012; Xi et al., 2015; Su et al., 2016; Omar et al., 2019). Transgenic maize plants expressing a chitinase gene showed enhanced resistance against corn borer (*Sesamia cretica*) (Osman et al., 2016). Insect chitinases have been established as biopesticides and transgenes in crop protection due to the inhibitory effects on the growth and development of insects. Not much research on insect chitinase has been done and lack of structural information on some insect chitinase has hampered the development of potential agrochemicals targeting insect chitinase. Better understanding of their structure and biochemistry will accelerate their usage in biotechnological processes.

### 2.2 Genome Editing

**FIGURE 4:**
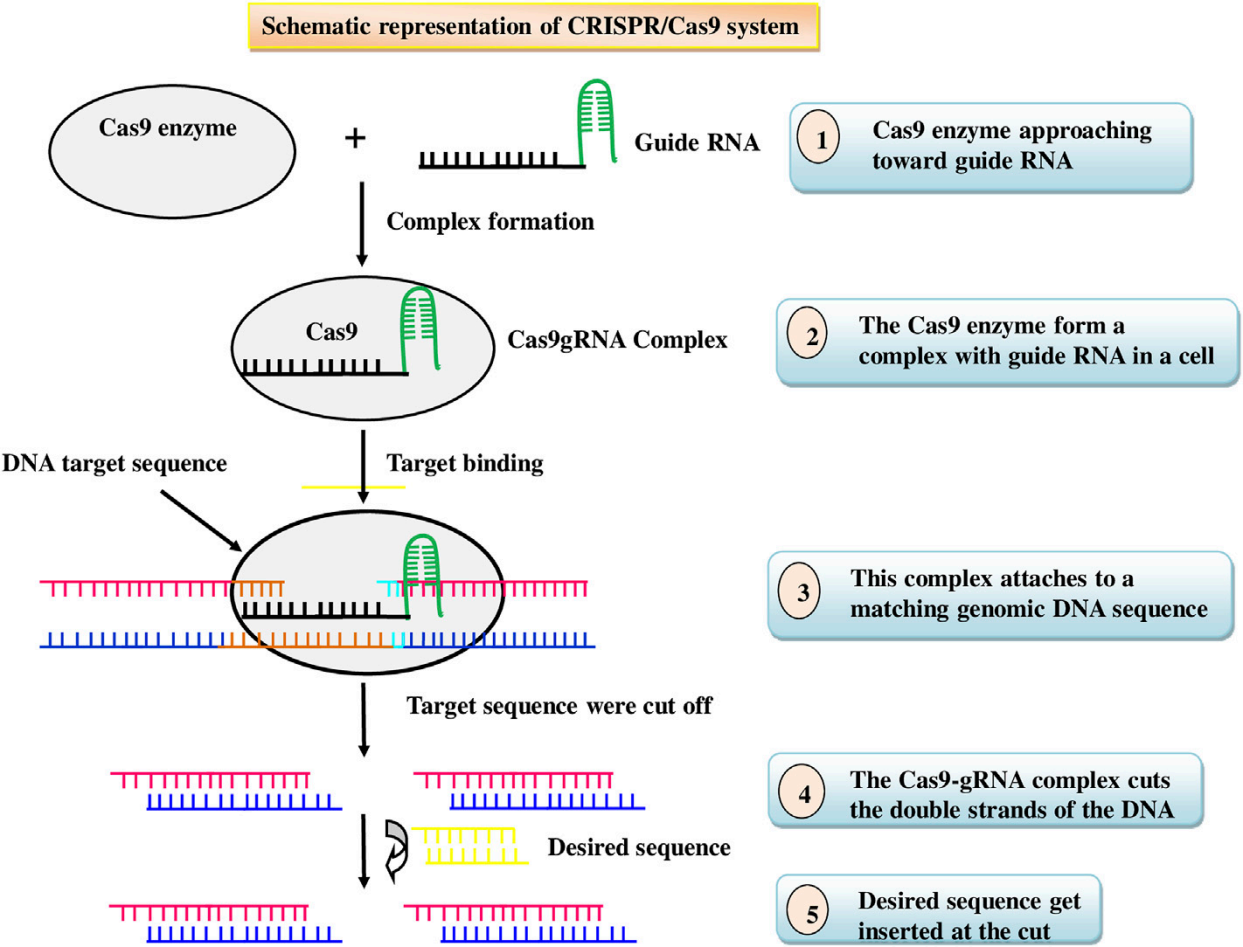
Schematic representation of CRISPR/Cas9 system.

Insects acquiring resistance to the *Bt* traits has posed a threat to agricultural productivity, prompting researchers to seek out novel, cost-effective, and environment friendly techniques to insect pest management, as well as ways to combat insect resistance. Nowadays, insect pest management tactic has shifted to gene editing, which is a newer and more advanced method (Anastacia Books, 2019). Gene editing, also called genome editing, is a technique that involves inserting, deleting, or replacing DNA bases in a specific target DNA sequence of the genome for effectively altering the function of a gene by using the cell’s natural mechanisms (Bortesi and Fischer 2015). It is one of the most widely used technologies in present-day science that empowers researchers to change/alter a living being’s DNA (Belfort and Bonocora, 2014). It is an emerging opportunity increasingly being used in insect pest management through expanding its possibilities and opportunities to enhance plant resistance to insect pests. Nucleases are used in these technologies to cut certain genomic target sequences. Two types of genome editing tools, comprising transcriptional activator-like effector nucleases (TALENs) and clustered regularly interspaced short palindromic repeats (CRISPR)/Cas9, are accessible and are frequently applied. Cas9 protein and single-guide RNAs (sgRNAs) which can be easily designed are the two main parts of the CRISPR/Cas9 system, while TALEN requires to be redesigned to target different loci each time. Cas9-mediated genome editing is achieved by a process: DNA cleavage followed by DNA repair (Figure 4). Furthermore, the CRISPR-Cas9 technology allows researchers to add, remove, replace, or regulate genes in a variety of animals, resulting in heritable, targeted modifications that were previously difficult to create (Ricroch et al., 2017). One of the most important practical advantages of CRISPR-Cas9 technology is multiplexing, or the introduction of double-stranded breaks (DSBs) at many locations in the genome that can be used to edit multiple genes simultaneously (Li et al., 2013). Thus, in recent times, CRISPR/Cas9 has emerged as a technically simple, newest, most effective, and an effective tool for developing insect pest resistance. It has been successfully used to prevent the accumulation of specific gene products in a variety of crops by either deleting the gene or inducing missense mutations in the target gene (Gao, 2021). Most polyphagous insects use the plant’s own volatiles, gustatory signs, visual appearance, oviposition sites, and collaborations to recognise host plants. (Larsson et al., 2004). Genome editing can be utilized to change plant volatile mixtures, which could be an alternative pest management strategy. However, caution should be taken to ensure that the alteration has no negative consequences for the beneficial insect population. In a study, the overproduction of anthocyanin pigmentation caused the transgenic tobacco plant’s leaves to turn red which deterred both the herbivores *Spodoptera litura* and *Helicoverpa armigera* due to change in leaf color (Malone et al., 2009). In an anotherstudy, CRISPR/Cas9 was usedto target six loci associated with tomato yield and efficiency in wild tomato *S. pimpinellifolium* (Zsögön et al., 2018). Although this wild tomato shows resistance to a numberof arthropod insects, including spider mites, and produces modest yields (Rakha et al., 2017). Ming et al. (2021) demonstrated that the CRISPR/Cas9 genome editing system is an effective tool for studying the function of SfABCC2, a *Cry1F* gene receptor that confers resistance to *S. frugiperda*. Insect-resistant rice plants with mutations in the cytochrome P450 gene CYP71A, which catalyses the conversion of tryptamine to serotonin, accumulated high levels of salicylic acid but lacked serotonin. (Lu et al., 2018). This could make genome editing more appealing than transgene stacking for the production of next-generation insect-resistant crops. Although CRISPR gene editing is an effective tool to combat insect pest problems as it has the capacity to alter the specific gene of interest. However, commercial use of CRISPR/Cas9 in insect pest management is still in its early stages. It has been extensively reformed for various applications in model animals, which may reveal potential insect applications. The list of insect pests engineered for pest management using CRISPR/Cas9 are given in Table 5.

**TABLE 5:** Insect engineered for pest management using CRISPR/Cas9.

Sr. No.	Target insects	Target gene	References
1	*Tribolium castaneum*	E-cadherin gene, *EGFP*	Gilles et al. (2015)
2	*Plutella xylostella*	Abdominal-A homeotic gene (*Pxabd-A*)	Huang et al. (2016)
3	*Drosophila melanogaster*	Chitin synthase 1	Douris et al. (2016)
4	*Agrotis ipsilo*	*Yellow-Y* Gene	Chen et al. (2016)
5	*Locusta migratoria*	Odorant receptor co-receptor (*Orco*) gene	Li et al. (2016)
6	*Spodoptera litura*	Abdominal-A (*Slabd-A*) gene	Bi et al. (2016)
7	*Spodoptera littoralis*	Olfactory receptor co-receptor (*Orco*) gene	Koutroumpa et al. (2016)
8	*Helicoverpa armigera*	*HaCad*	Wang et al. (2016)
9	*Spodoptera exigua*	Ryanodine receptor	Zuo et al. (2017)
10	*Ceratitis capitata*	Eye Pigmentation Gene White Eye (*We*)	Meccariello et al. (2017)
11	*Helicoverpa armigera*	*Tetraspainin*	Jin et al. (2018)
12	*Plutella xylostella*	*PxABCC2, PxABCC3*	Guo et al. (2019)
13	*Helicoverpa armigera*	α-6- nicotinic acetylcholine receptor (*nAchR*)	Zuo et al. (2020)
14	*Rhopalosiphum padi*	*ß-1-3glucanase* in maize	Kim et al. (2020)
15	*Ostrinia furnacalis*	*ABCC2*	Wang et al. (2020a), Wang et al. (2020b)

### 2.3 RNA Interference for Plant Resistance to Insect

**FIGURE 5:**
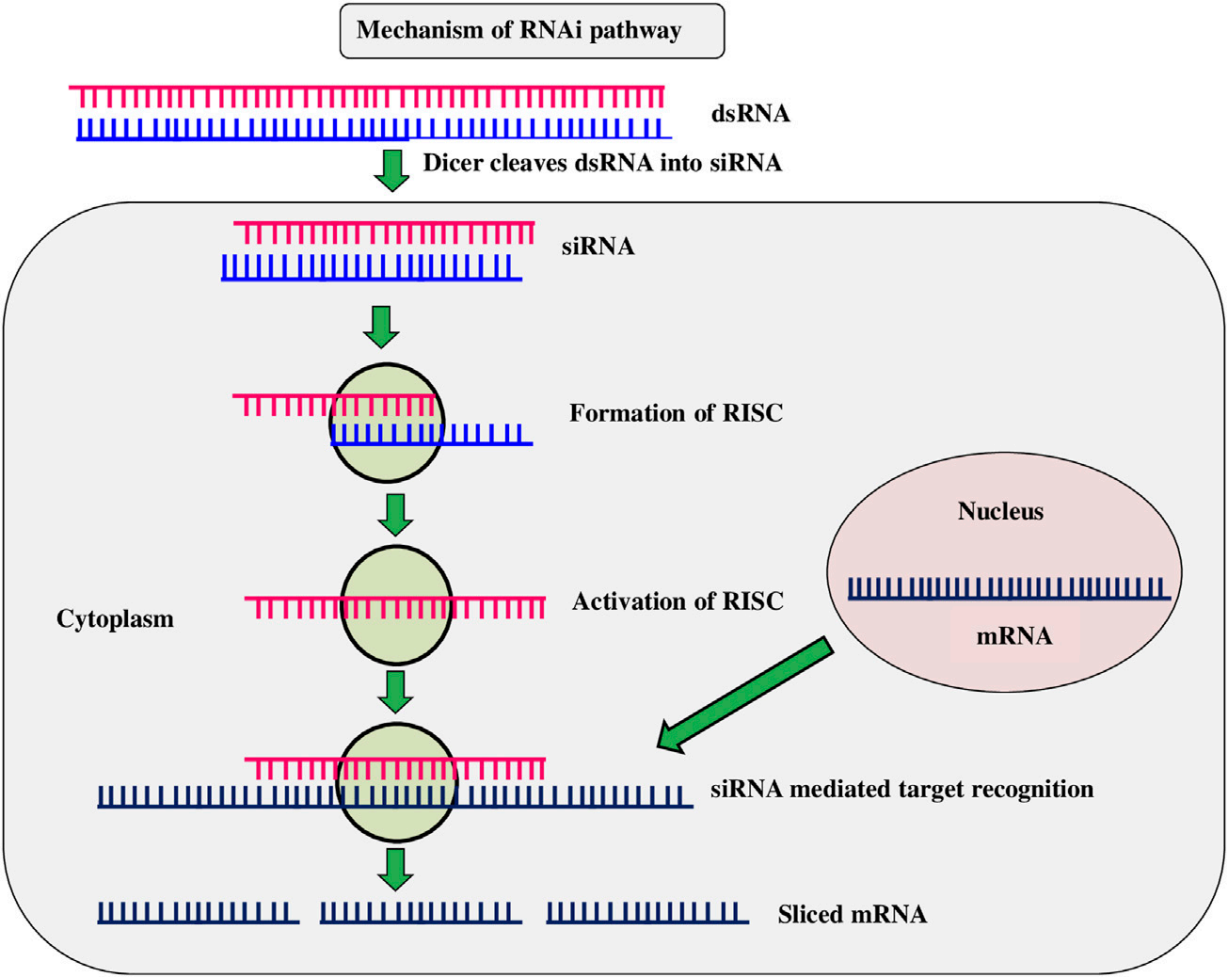
RNA interference (RNAi).

RNAi is a method of suppressing gene expression by suppressing specific sequences and is known by various names, including co-concealment, post-transcriptional gene silencing (PTGS), and suppressing. It is an advancement of novel gene silencing mechanisms triggered by double-stranded RNA at the cellular level. (Figure 5). When a double-stranded RNA (dsRNA) is injected into a cell, it makes undesirable genes to be repressed (Kamthan et al., 2015). The RNAi strategy for pest control is based on ingestion of double-stranded RNA (dsRNA) into the target pest system. After ingestion, dsRNA expresses either through hairpin or by other different means and spread throughout the insect system (Katoch et al., 2013). Transgenic *Bt* toxins are mostly effective against Lepidoptera and Coleoptera larvae, by acting in the mid-gut of susceptible target insects, leaving other insect orders unmanaged. The RNAi technique was expected to be able to control a wider range of insects, especially sap-sucking insects, which transgenic crops had failed to control. It also opens up new possibilities for eco-friendly insect pest control in agricultural crop plants (Mamta and Rajam 2017). dsRNAs are often utilized in plants to interfere with specific gene silence in order to develop disease resistance through genetic changes. Resistance to *C. suppressalis* was provided by rice knockdown lines TT51 (cryAb and cry1Ac) and T1C-19 (cry1Ac) with two aminopeptidase N genes (APN1 and APN2) (Qiu et al., 2017). Western corn rootworm (*D. virgifera*) fertility and larval feeding were reduced when the *dvvgr* and *dvbol* genes were silenced in maize (Niu et al., 2017). The use of a dsRNA/nano carrier formulation to target the TREH, ATPD, ATPE, and CHSI genes resulted in a greater proportion of soybean aphid (*Aphis glycines*) mortality (Yan et al., 2020). Transgenic cotton lines generated by combining *Bt* toxin with RNAi caused inhibition of juvenile hormone methyl transferase (JHMT) in *H. armigera*. (Ni et al., 2017). Knockdown of acetylcholine esterase gene (*AChE*) in rice lines resulted in reduced larval length and weight of yellow stem borer within 15 days (Kola et al., 2019). Ingestion of double-stranded (ds) RNAs in an artificial diet causes RNA interference in several coleopteran species, including the western corn rootworm (WCR) *Diabrotica virgifera virgifera*, which results in larval stunting and mortality. (Baum et al., 2007). The RNAi mechanism was tested by the spraying of dsRNAs in maize, resulting in gene knockdown and increased insect mortality rates in piercing, sucking, and stem borer insects (Li et al., 2015). Mamta et al. (2016) found that HI-RNAi produced induced death and developmental abnormalities in *H. armigera* larval, pupal, and adult stages when the chitinase gene (HaCHI) was silenced to establish resistance in tobacco and tomato. Tomato plants with a dsRNA targeting a gene encoding a phenolic glucoside malonyltransferase, which detoxifies phenolic glycosides, were recently found to be completely resistant to the tobacco whitefly, *Bemisia tabaci* (Xia et al., 2021). The Colorado potato beetle (Zhang et al., 2015), tobacco whitefly and tobacco hornworm, *Manduca sexta* (Burke et al., 2019), were all killed by chloroplast-expressed dsRNAs (Dong et al., 2020). Di Lelio et al. (2022) reported that when *Spodoptera littoralis* larvae eat tobacco plants expressing a dsRNA targeting the Sl 102 immune gene, the gene get silenced (et al.). RNAi technology is effective in knocking down target genes in a variety of insect orders, including *Diabrotica v. virgifera*, maize rootworm larvae (Khajuria et al., 2015). These findings suggest that to generate insect-resistant plants, RNAi is one of the most effective methods. However, the technology is currently being investigated, and its existing limitations make it less viable as an insect pest management strategy (Anastacia Books, 2019).

The most challenging task of this technology is allowing for efficient dsRNA uptake by the insect. The generation and transport of dsRNAs have been proven in two ways. The first is HIGS (host induced gene silencing), which involves the transgenic expression of dsRNAs derived from the crop genome. In this method, insects supposed to feed on the crop will eat the dsRNA. In the second strategy, dsRNAs are synthesized in high concentrations and applied to insect-infested crops as a foliar spray. Spray-induced gene silencing is the name for this method (SIGS). The target genes will be silenced in both approaches in the target species (Christiaens et al., 2020a). Many challenges are still there. The inherent ability of RNA, while ensuring that dsRNAs don’t persist in the environment, is destructive in unfavorable environmental conditions and restricts opportunities for SIGS approaches (Bramlett et al., 2020). Similarly, while some insects quickly take up dsRNA, resulting in high death rates, other species have low dsRNA take-up and nuclease degradation, resulting in inefficient outcomes. (Christiaens et al., 2020b; Shaffer, 2020). Success is also dependent on whether sufficient amount of dsRNA accumulate in the tissues on which the insects feed. The development of novel spray formulations, many of which use nanomaterials, is being used to address dsRNA stability and uptake in ongoing research. (Christiaens et al., 2020b). Several studies on insect pest management using the RNAi tool are shown in Table 6.

**TABLE 6:** Transgenic crops for insect resistance through RNA interference.

Sr. No.	Target insects	Silenced gene	Target crop	References
1	D*iabrotica virgifera virgifera* LeConte	Suppression of target mRNA	Maize	Baum et al. (2007)
2	*Diabrotica v. virgifera*	hunchback (hb) and brahma (brm) gene	Maize	Khajuria et al. (2015)
3	*Leptinotarsa decemlineata*	*β-actin* gene	Potato	Zhang et al. (2015)
4	Lepidopteran	dsRNA-Spray	Maize	Li et al. (2015)
5	*H. armigera*	Chitinase gene-*HaCHI*	Tomato, Tobacco	Mamta et al. (2016)
6	*C. suppressalis*	Aminopeptidase N genes *APN1+APN2*	Rice	Qiu et al. (2017)
7	*Leguminivora glycinivorella*	SpbP0-dsRNA	Soyabean	Meng et al. (2017)
8	*Helicoverpa armigera*	Juvenile hormone methyl transferase (JHMT)	Cotton	Ni et al. (2017)
9	D*iabrotica virgifera virgifera* LeConte	*Dvvgr, dvbol*	Maize	Niu et al. (2017)
10	*Leptinotarsa decemlineata*	*ECR* gene	Potato	Hussain et al. (2019)
11	*Scirpophaga incertulas*	*AchE*-Acetylcholine esterase	Rice	Kola et al. (2019)
12	*Manduca sexta*	v*-ATPaseA* gene	Tobacco	Burke et al. (2019)
13	*Bemisia tabaci*	*BtACTB gene*	Tobacco	Dong et al. (2020)
14	*Aphis glycines*	*TREH, ATPD, ATPE, CHSI*	Soyabean	Yan et al. (2020)
15	*Bemisia tabaci*	Phenolic glucoside malonyltransferase	Tobacco	Xia et al. (2021)
16	*Spodoptera littoralis*	*Sl 102 immune* gene	Tobacco	Di Lelio et al. (2022)

### 2.4 Marker-Assisted Selection

**FIGURE 6:**
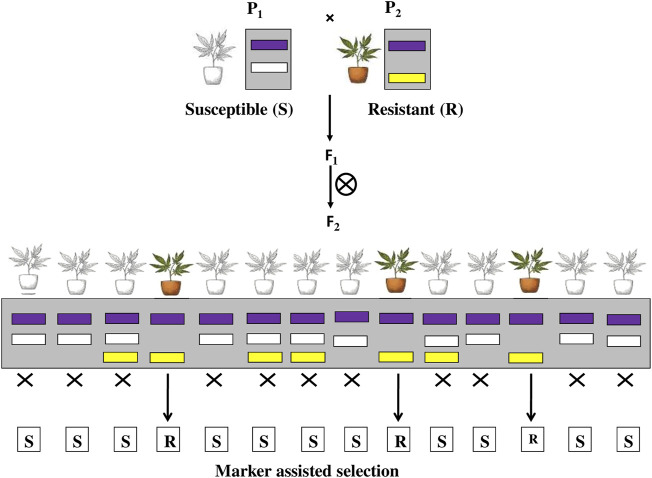
Marker assisted selection and its application in insect pest management.

The use of molecular markers to assist phenotypic selections in crop improvement is known as marker-assisted selection (MAS). It involves selecting individuals based on their marker pattern (genotype) rather than their observable traits (phenotype) as shown in Figure 6. There are various types of molecular markers, such as single nucleotide polymorphism (SNP), have been recognised and have shown great promise in enhancing the efficiency and accuracy of conventional plant breeding. Molecular marker techniques are the most advanced method for transferring desired genes into desired crop plants in the required combination. It is the most widely used molecular techniques, and their application is a novel opportunity for increasing the yield of crop (Das et al., 2017). MAS studies showed introgression of *Bph14* and *Bph15* through molecular marker-assisted selection (MAS) to enhance the resistance in Minghui 63 and its derived hybrids against BPH (Hu et al., 2012). Resistance to bacterial blight (BB) and brown planthopper (BPH) was achieved in Yuehui9113 and F1 hybrids by pyramiding one BB resistance gene (*Xa21*) and two BPH resistance genes (*Bph14 and Bph15*) in Yuehui9113 using a marker-assisted backcrossing (MABC) strategy combined with phenotypic selection (He et al., 2019). Rice line, ASD7 expressing a BPH resistance gene *bph2* when crossed to a susceptible cultivar C418, a japonica restorer line and evaluated through marker-assisted selection (MAS) exhibited significantly higher resistance against brown plant hopper *Nilaparvata lugens,* one of the most destructive pests of rice crop (Li-Hong et al., 2006). Liu et al. (2016) investigated that the pyramiding of two brown plant hopper resistance genes *Bph3* and *Bph27* (t), into elite rice cultivars through marker-assisted pyramiding showed significantly enhanced resistance against BPH and reduction in the yield loss caused by BPH. Shabanimofred et al. (2015) developed rice cultivars through marker-assisted selection (MAS) that provided resistance in rice against biotypes 2 and 3 of brown planthopper (BPH). Sharma et al. (2004) used marker-assisted pyramiding to successfully construct the *Bph1* and *Bph2* resistance genes on rice chromosome 12 to provide resistance against rice BPH. et al. As a result, using MAS to improve pest resistance would be very beneficial. There are various advantages of using MAS to enhance selection efficiency of insect resistant plants 1) It can be performed on seedling material, 2) less affected by environmental conditions, 3) MAS may be cost effective and faster than conventional phenotypic assays, 4) multiple markers can be evaluated using the same DNA sample etc. But the potential drawbacks of MAS are 1) Recombination between the marker and the gene of interest may occur, leading to inaccurate results 2) Incorrect estimates of QTL locations and effects may result in slower progress than expected, 3) Markers developed for MAS in one population may not be transferrable to other populations.

### 2.5 Anther Culture

Anther culture is a technique by which immature pollen is allowed to divide and grow into tissue (either callus or embryonic tissue) with the intention of generating haploids (plants with a N chromosome number). In this process, pollen-containing anthers are separated from a flower and placed in a suitable growing medium. An artificial medium could be used to culture anther or pollen grain *in vitro*. Anther can produce a callus, shoot, root, and eventually the entire plant in an artificial medium. All of the plants that are grown are haploid. It is the most viable and effective way for rapidly producing homozygous haploid plants. This method can hasten the development of a homozygous population of insect-resistant plants. Rice anther culture lines, 952836, 953508, 953509, 953510, 953511, 953527 and 953541 showed moderate level of tolerance to the rice water weevil, *Lissorhoptrus oryzophilus* Kuschel (N’guessan et al., 1994). Park et al. (2014) develop multi-resistant rice lines using anther culture for providing resistance against bacterial blight, rice stripe virus and brown planthopper.

### 2.6 Embryo Culture

Embryo culture is a technique in which immature or mature zygotic embryo is recovered without injury which normally aborts. These embroys are further cultured on the artificial nutrient media under an aseptic environment to get a vigor and viable plant through a successful ontogeny process. The standardization of the nutrient medium is required for induction of embryogenesis and seedling development. Generally wild species are often more resistant to insect pests. Wide hybridization has been used to transfer genes conferring insect-resistant from wild species to cultivated plants. It has been observed that such hybridization leads to the production of abnormal inter-specific hybrid embryos, which can be rescued using embryo culture technique. Jaiswal et al. (2018) has reported studies of insect resistant genes transfer from wild to cultivated species in wheat, rice, peanut, lettuce and cotton using embryo culture technique.

### 2.7 Protoplast Fusion

Protoplasts are plant cells of which cell walls are taken out and the cytoplasmic membrane is the peripheral layer. Protoplast can be isolated by digesting the cell wall with specific lytic enzymes. Protoplast fusion is a physical phenomenon, wherein at least two protoplasts come together and stick with each other either spontaneously or in presence of fusion-inducing agents. By protoplast fusion, it is feasible to transfer a few desirable genes from one species to another. For example, pest resistance characteristics may be present in one of two species that cannot be sexually hybridized. In this situation, protoplast fusion may result in the formation of a hybrid between two species. Protoplasts can be cultivated in an artificial medium, and some of them will grow into full-fledged plants. Thus the plants produced may be carrying the resistant traits. Also, it is the only means of combining two cytoplasmically inherited characteristics in a single genotype. Protoplast derived clones produced by Mexican wild species and cultivated potato species using protoplast fusion system expresses a significant level of resistance to both Colorado potato beetle and potato late blight (Chen et al., 2008).

### 2.8 Somaclonal Variation

Insect-resistant varieties can be selected through somaclonal variation. These can be chosen using the procedures below. 1) High-yielding varieties’ calli or cell suspensions were cultured for numerous or long-term cycles, 2) long-term cell lines were regenerated into plants, and 3) the regenerated plants were tested against target insects. In field plots, about 2000 sugarcane seedlings were tested for resistance to the sugarcane borer under artificial and natural infestations and it was found that some somaclones were reported to be resistant to sugarcane borer. The same process was utilized to develop sorghum somaclones, resistant to the autumn armyworm. Diawara et al. (1996) reported that somaclonal lines K-26 [1], K-180 [3]2 and K-128 exhibited improved resistant to celery major insect, *Spodoptera exigua*.

Although the above-mentioned plant tissue culture techniques like anther culture, embryo culture, protoplast fusion, and somaclonal variations proved to be more efficient in the development of plants resistant to various insect pests. However, these techniques are not widely used due to some potential drawbacks, such as high costs, the production of harmful secondary metabolites that kill the desired insect-resistant plants, the medium required for growth is not known, and so on. Also, due to advancements in technology, which are more efficient, quick, and reliable, these methods are no longer used.

## 3 Conclusion and Future Outlooks

Insects are the major concern for declining the agricultural production. To cope with the problem of insect pests, farmers are more inclined to the use of chemical insecticides as these provide a quick solution to the problem. The rapidly increasing awareness of the human and animal health issues as well as environmental impacts, of indiscriminate use of pesticide has offered new incentive to the potential alternative pest-control methods. In this perspective, host plant resistance is an environmentally friendly control method that is an important part of IPM (Integrated pest management) programmes. The development of insect-resistant varieties offers a stable and cumulative effect on the pests’ population and has no harmful effect on the environment. The identification of insect pest resistant sources in various crops has made significant progress. However, development of insect resistant crop varieties through conventional methods is slow and difficult to attain due to the entanglement of quantitative traits at multiple loci. New opportunities in the form of newer biotechnological tools have opened new ways of pest control and offers great opportunities to develop a sustainable, multi-mechanistic resistance to insect pests. Biotechnological approaches are now being used to develop novel plant resistance characteristics that provided excellent protection against invasive and destructive crop pests in a variety of crops by utilization of novel molecules, exploiting insecticidal genes and changing the level and pattern of expression of genes. Many insect-resistant plants have been developed as a result of biotechnology like corn, rice, cotton, canola, soybean, tobacco, apple, potato etc. With the advent of several tools of biotechnology such as genome editing, genetic transformation, anther culture, embryo culture, protoplast fusion, somaclonal variation, and marker-assisted selection will accelerate the development of insect-resistant crops now and in the future. By expressing bacterial delta-endotoxins, vegetative insecticidal proteins, and other plant qualities like lectins, protease inhibitors, etc., hereditary designing will guide towards the development of insect-resistant crops at much faster rate. Furthermore, RNA interference and genome editing by CRISPR/Cas9 offer novel approach to the production of insect-resistant crops. Therefore, biotechnology have come as a boon in tackling global pest problem, contributing to the development of noval insect resistant crop plants that have proven to be cost effective, pesticide-resistant, and environmentally safe. Despite the utilization of modern technology in crops to achieve resistance to a variety of insect pests, some agricultural pests frequently develop resistance to insecticidal toxins, wreaking havoc on crop productivity. The obstacles of understanding plant-insect interactions should be addressed by the research groups. To develop plants resistant to insects advances like RNAi and CRISPR techniques can be used to silence/edit sensitive or negative regulatory alleles of plant immune genes. New advancements that give more viable solutions for arising pests can improve and supplement the perseverance of plant-resistant elements. However, before the commercialization of an insect pest resistant transgenic crop variety, it is pertinent to study the potential impacts on environment specifically on non-target organisms. Also, the benefits and hazards associated with the adoption of insect-resistant crops, particularly for developing nations and resource-poor smallholder farmers, should be considered prior to carry out such initiatives. No doubt, biotechnology has opened the door to a plethora of novel ways for controlling insect pests, many of these products will necessitate regulatory frameworks that may not currently exist for certain products or in some places. They will also require the support of producers and consumers, which will necessitate open conversations, including the potential for new technology to make a significant contribution to societal change. In short it can be concluded that biotechnology exhibits unique applications of science that can be used for the welfare of society through the development of crops with improved nutritional quality, resistance to pests and diseases, and low cost of production. Biotechnology, in this context, is an aspect of science that, if used with caution and ethics, has the potential to offer substantial benefits.
