# Supplementary material for: Biotechnological Approaches for Host Plant Resistance to Insect Pests
Source: Front Genet. 2022 Jun 2;13:914029. doi: 10.3389/fgene.2022.914029 (PMC9201757; doi:10.3389/fgene.2022.914029)
Supplement: Supplementary file 1 [file DataSheet1.doc]

**Table 1. Transgenic crops carrying *Bt* genes for insect resistance**

| **S. No.** | **Target insects** | **Transgene** | **Target crop** | **Reference** |
| --- | --- | --- | --- | --- |
| 1 | *Chilo suppressalis*, *Cnaphalocrocis medinalis* | *cry 1a(b)* | Rice | Fujimoto *et al*. (1993) |
| 2 | *Scirpophaga incertulas* & *Chilo suppressalis* | *cry 1 a(b)* | Rice | Wunn *et al*. (1996) |
| 3 | *Scirpophaga incertulas*, *Cnaphalocrocis medinalis* | *cry 1 a(b)* | Rice | Ghareyazie *et al*. (1997) |
| 4 | *Scirpophaga incertulas* | *cry 1a(c)* | Rice | Nayak *et al*. (1997) |
| 5 | *Scirpophaga incertulas* | *cry 1a(b)/ cry1a(c)* | Rice | Tu *et al*. (2000) |
| 6 | *Cnaphalocrocis medinalis*, *Scirpophaga incertulas* | *cry 2a/ cry 1a(c)* | Rice | Maqbool *et al*. (2001) |
| 7 | *Helicoverpa armigera* | *cry1Ab+NptII* | Cotton | Khan *et al*. (2011) |
| 8 | *Heliothis sp.* | *cry1Ab* | Cotton | Ali Khan *et al*. (2013) |
| 9 | *Helicoverpa armigera* | *cry2AX* | Cotton | Sakthi *et al*. (2015) |
| 10 | *Helicoverpa armigera* | *cry1AC+ cry2Aa* | Pigeon pea | Ghosh *et al.* (2017) |
| 11 | *Helicoverpa armigera* | *cryIIAa* | Chickpea | Sawardekar *et al*. (2017) |
| 12 | *Cnephalocrosis medinalis* | *cry2A* | Rice | Gunasekara *et al*. (2017) |
| 13 | *Tuta absoluta* | *cry1Ac* | Tomato | Selale *et al*. (2017) |
| 14 | *Anthamous grandis* | *cry1Aa* | Cotton | Ribeiro *et al*. (2017) |
| 15 | *Helicoverpa armigera* | *cry2Aa* | Pigeon pea | Baburao and Sumangala (2018) |
| 16 | *Helicoverpa armigera* | *cry2Aa* | Pigeon pea | Singh *et al*. (2018) |
| 17 | *Scirpophaga incertulas*, *Cnaphalocrocis medinalis* | *cry2AX1* | Rice | Rajadurai *et al*. (2018) |
| 18 | *Spodoptera litura* | *cry1Aa* | Sweet Potato | Zhong *et al*. (2019) |
| 19 | *Spodoptera litura* | *cry1AC+cry2Ab* | Cotton | Siddiqui *et al*. (2019) |
| 20 | *Holtrichia panallele* | *cry 8 like* | Soyabean | Qin *et al*. (2019) |
| 21 | *Achaea janata, Spodoptera litura* | *cry1AC* | Castor | Muddanuru *et al*. (2019) |
| 22 | *Helicoverpa armigera* | *cry2AX1* | Cotton | Jadhav *et al*. (2020) |
| 23 | *Tuta absoluta* | *cry1Ab* | Tomato | Soliman *et al*. (2021) |

**Table 2. Expression of *VIP* genes for insect resistance**

| **S. No.** | **Target insects** | **Transgene** | **Target crop** | **Reference** |
| --- | --- | --- | --- | --- |
| 1 | *Heliothis. zea* and *H. virescens* | *Vip3A+cry1Ab* | Cotton | Bommireddy *et al.* (2011) |
| 2 | *Maruca vitrata* | *Vip3Ba1* | Cowpea | Bett *et al.* (2017) |
| 3 | *Helicoverpa armigera* | *Vip3AcAaa (Vip3Aa1+Vip3Ac1)* | Cotton | Chen *et al.* (2018) |
| 4 | *Chilo infuscatellus* | *Vip3A* | Sugarcane | Riaz *et al.* (2020) |

**Table 3. Expression of lectin genes for insect resistance**

| **Sr. no.** | **Target insects** | **Transgene** | **Target crop** | **Reference** |
| --- | --- | --- | --- | --- |
| 1 | Sap sucking pests | Snowdrop lectin (*Galanthus nivalis* agglutinin; GNA) | Rice | Sudhakar *et al*. (1998) |
| 2 | *Sitobion avenae* | Snowdrop lectin (*Galanthus nivalis* agglutinin; GNA) | Wheat | Stoger *et al*. (1999) |
| 3 | *Eoreuma loftini* (Dyar) and *Diatraea saccharalis* | Snowdrop lectin (*Galanthus nivalis* agglutinin; GNA) | Rice, Sugarcane | Setamou *et al*. (2002) |
| 4 | *Lacanobia oleracea* | GNA-neuropeptide-allatostatin | Tomato | Fitches *et al*. (2004) |
| 5 | *Lacanobia oleracea* | GNA-lepidopteran-specific toxin (ButalT) | Tomato | Trung *et al*. (2006) |
| 6 | *Nilaparvata lugens and Myzus persicae)* | GNA-spider-venom toxin I (SFI1) | Rice and  Potato | Down *et al*. (2006); |
| 7 | *Aedes aegypti* eggs and larvae | WSMoL (water-soluble *M. oleifera* lectin) | - | Coelho *et al*. (2009); Santos *et al*. (2012, 2020) |
| 8 | *Nilaparvata lugens, Sogatella furcifera and Nephotettix nigropictus* | *Allium sativum* leaf agglutinin (ASAL) and *Galanthus nivalis* lectin (GNA) | Rice | Bharathi *et al*. (2011) |
| 9 | *Nasutitermes corniger* workers and soldiers | Endoglucanase, phosphatases, b-glucosidase, and trypsin | - | de Albuquerque *et al*. (2012) |
| 10 | *Sitophilus zeamais* | *M. urundeuva* leaf lectin (MuLL) | Stored grains | Napoleao *et al*. (2013) |
| 11 | *Myzus persicae* and *Sitobion avenae* | Hv1a/GNA | Potato | Nakasu *et al.* (2014) |
| 12 | *Myzus persicae* | *Galanthus nivalis* agglutinin (GNA) | Potato | Mi *et al*. (2017) |
| 13 | *Lipaphis erysimi* | Lentil lectin (LL) and Chickpea protease inhibitor (CPPI) genes | Transgenic *Brassica juncea* | Rani *et al*. (2017b) |
| 14 | *Sitophilus zeamais* | *Schinus terebinthifolius* leaf lectin (SteLL) | Stored grains | de Santana Souza *et al*. (2018) |
| 15 | *Lipaphis erysim*i | *Colocasia esculenta* tuber agglutinin (CEA)+ *Galanthus nivalis* agglutinin (GNA) | Mustard | Das *et al*. (2018) |
| 16 | *Metopolophium dirhodum*, *Schizaphis graminum*, *Rhopalosiphum padi*, and *Sitobion avenae* | *Pinellia pedatisecta* agglutinin (PPA) | Wheat | Duan *et al.* (2018) |
| 17 | *Aphis gossypii* and *Spodoptera litura* | Insect gut binding lectin from *Sclerotium rolfsii* | Cotton | Vanti *et al*. (2018) |
| 18 | *Callosobruchus chinensis* | Arcl on APA locus from *Phaeselous vulgaris* | Cowpea | Grazziotin *et al*. (2020) |
| 19 | *Sitophilus. zeamais* | *Microgramma vacciniifolia* rhizome lectin (MvRL) | Stored grains | de Albuquerque *et al*. (2020) |
| 20 | *Sitophilus zeamais* | Water-soluble *Moringa oleifera* lectin (WSMoL) | Stored grains | de Oliviera *et al*. (2020) |
| 21 | *Callosobruchus chinensis* | Arcelin | Common bean | Hilda *et al*. (2022) |
| 22 | *Callosobruchus chinensis* | Arcelin-5, Leucoagglutinin, Erythroagglutinin | common bean | Caroline *et al*. (2022) |
| 23 | *Sitophilus oryzae* | *Polygonum persicaria* L. (PPA) Lectin | Stored grains | Khoobdel *et al* (2022) |

Table 4. Fusion proteins for insect resistance in crop plants

| **Sr. no.** | **Target insects** | **Transgene** | **Target crop** | **Reference** |
| --- | --- | --- | --- | --- |
| 1 | *Scirpophaga incertulas*, *Cnaphalocrocis medinalis* | *cry2AX1 (cry2Aa+cry2Ac)* | Rice | Chakraborty *et al*. (2016) |
| 2 | *Lygus spp.* | *cry51Aa2* | Cotton | Gowda *et al*. (2016) |
| 3 | *Spodoptera exigua, Harmonia axyridis* | *cry1Ab/cry2Aj* | Maize | Chang *et al.* (2017) |
| 4 | *Spodoptera litura, Ostrinia nubialis* | *cry1Be+cry1Fa* | Cotton | Meade *et al.* (2017) |
| 5 | *Lipaphis erysimi* | Lentil lectin (LL) and chickpea protease inhibitor (CPPI) genes | *Brassica juncea*- mustard | Rani *et al*. (2017b) |
| 6 | *Scirpophaga incertulas*, *Cnaphalocrocis medinalis, Nilaparvata lugens* | *cry1AC+ASAL* | Rice | Boddupally *et al*. (2018) |
| 7 | *Ostrinia furnacalis,* *Cnaphalocrocis medinalis* | *cry1Ab+vip3A* | Rice | Xu *et al*. (2018) |
| 8 | *Chilo suppressalis* | *cry2Aa+cry1Ca* | Rice | Qiu *et al*. (2019) |
| 9 | *Helicoverpa armigera, Spodoptera litura* | *cry2Ab+cry1F+cry1AC* | Cotton | Katta *et al*. (2020) |
| 10 | *Scirphophaga excerptalis* | *cry2Aa+cry1Ca, cry1Ab+cry1Ac* | Sugarcane | Koerniati *et al*. (2020) |

**Table 5. Insect engineered for pest management using CRISPR/Cas9**

| **Sr. no.** | **Target insects** | **Target gene** | **Reference** |
| --- | --- | --- | --- |
| 1 | *Tribolium castaneum* | E-cadherin gene, *EGFP* | Gilles *et al*. (2015) |
| 2 | *Plutella xylostella* | Abdominal-A homeotic gene (*Pxabd-A*) | Huang *et al*. (2016) |
| 3 | *Drosophila melanogaster* | Chitin synthase 1 | Douris *et al*. (2016) |
| 4 | *Agrotis ipsilo* | *Yellow-Y* Gene | Chen *et al*. (2016) |
| 5 | *Locusta migratoria* | Odorant receptor co-receptor *(Orco)* gene | Li *et al*. (2016) |
| 6 | *Spodoptera litura* | Abdominal‐A (*Slabd‐A*) gene | Bi *et al*. (2016) |
| 7 | *Spodoptera littoralis* | Olfactory receptor co-receptor (*Orco*) gene | Koutroumpa *et al*. (2016) |
| 8 | *Helicoverpa armigera* | *HaCad* | Wang *et al*. (2016) |
| 9 | *Spodoptera exigua* | Ryanodine receptor | Zuo *et al*. (2017) |
| 10 | *Ceratitis capitata* | Eye Pigmentation Gene White Eye (*We*) | Meccariello *et al*. (2017) |
| 11 | *Helicoverpa armigera* | *Tetraspainin* | Jin *et al*. (2018) |
| 12 | *Plutella xylostella* | *PxABCC2, PxABCC3* | Guo *et al*. (2019) |
| 13 | *Helicoverpa armigera* | α-6- nicotinic acetylcholine receptor (*nAchR*) | Zuo *et al*. (2020) |
| 14 | *Rhopalosiphum padi* | *ß-1-3glucanase* in maize | Kim *et al*. (2020) |
| 15 | *Ostrinia furnacalis* | *ABCC2* | Wang *et al*. (2020a, b) |

**Table 6. Transgenic crops for insect resistance through RNA interference**

| **Sr. no.** | **Target insects** | **Silenced gene** | **Target crop** | **Reference** |
| --- | --- | --- | --- | --- |
| 1 | D*iabrotica virgifera virgifera* LeConte | Suppression of target mRNA | Maize | Baum *et al*. (2007) |
| 2 | *Diabrotica v. virgifera* | hunchback (hb) and brahma (brm) gene | Maize | Khajuria *et al*. (2015) |
| 3 | *Leptinotarsa decemlineata* | *β-actin* gene | Potato | Zhang *et al*. (2015) |
| 4 | Lepidopteran | dsRNA-Spray | Maize | Li *et al*. (2015) |
| 5 | *H. armigera* | Chitinase gene-*HaCHI* | Tomato, Tobacco | Mamta *et al*. (2016) |
| 6 | *C. suppressalis* | Aminopeptidase N genes *APN1+APN2* | Rice | Qiu *et al*. (2017) |
| 7 | *Leguminivora glycinivorella* | SpbP0-dsRNA | Soyabean | Meng *et al*. (2017) |
| 8 | *Helicoverpa armigera* | Juvenile hormone methyl transferase (JHMT) | Cotton | Ni *et al*. (2017) |
| 9 | D*iabrotica virgifera virgifera* LeConte | *Dvvgr, dvbol* | Maize | Niu *et al*. (2017) |
| 10 | *Leptinotarsa decemlineata* | *ECR* gene | Potato | Hussain *et al*. (2019) |
| 11 | *Scirpophaga incertulas* | *AchE*-Acetylcholine esterase | Rice | Kola *et al*. (2019) |
| 12 | *Manduca sexta,* | v*-ATPaseA* gene | Tobacco | Burke *et al*. (2019) |
| 13 | *Bemisia tabaci* | *BtACTB gene* | Tobacco | Dong *et al*. (2020) |
| 14 | *Aphis glycines* | *TREH, ATPD, ATPE, CHSI* | Soyabean | Yan *et al*. (2020) |
| 15 | *Bemisia tabaci* | Phenolic glucoside malonyltransferase | Tobacco | Xia *et al*. (2021) |
| 16 | *Spodoptera littoralis* | *Sl 102 immune* gene | Tobacco | Di Lelio *et al*. (2022) |

**References:**

Ali Khan, G., Bakhsh, A., Ghazanfar, M., Riazuddin, S., & Husnain, T. (2013). Development of transgenic cotton lines harboring a pesticidal gene (cry1Ab). *Emirates Journal of Food & Agriculture (EJFA)*, 25(6).https://doi.org/10.9755/ejfa.v25i6.13133.

Baburao, T. M., & Sumangala, B. (2018). Development and molecular characterization of transgenic Pigeon pea carrying cry2Aa for pod borer resistance. *J. Pharm. Phytochem*, 75, 1581-1585.

Baum, J. A., Bogaert, T., Clinton, W., Heck, G. R., Feldmann, P., Ilagan, O., ... & Roberts, J. (2007). Control of coleopteran insect pests through RNA interference. *Nature biotechnology*, 25(11), 1322-1326. <https://doi.org/10.1038/nbt1359>

Bett, B., Gollasch, S., Moore, A., James, W., Armstrong, J., Walsh, T., ... & Higgins, T. J. (2017). Transgenic cowpeas (*Vigna unguiculata* L. Walp) expressing *Bacillus thuringiensis* Vip3Ba protein are protected against the Maruca pod borer (*Maruca vitrata*). *Plant Cell, Tissue and Organ Culture (PCTOC)*, 131(2), 335-345. https://doi.org/10.1007/s11240-017-1287-3.

Bharathi, Y., Kumar, S. V., Pasalu, I. C., Balachandran, S. M., Reddy, V. D., & Rao, K. V. (2011). Pyramided rice lines harbouring *Allium sativum* (asal) and *Galanthus nivalis* (gna) lectin genes impart enhanced resistance against major sap-sucking pests. *Journal of Biotechnology*, 152(3), 63-71. https://doi.org/10.1016/j.jbiotec.2011.01.021.

Bi, H. L., Xu, J., Tan, A. J., & Huang, Y. P. (2016). CRISPR/Cas9‐mediated targeted gene mutagenesis in *Spodoptera litura*. *Insect Science*, 23(3), 469-477. https://doi.org/10.1111/1744-7917.12341.

Boddupally, D., Tamirisa, S., Gundra, S. R., Vudem, D. R., & Khareedu, V. R. (2018). Expression of hybrid fusion protein (Cry1Ac:: ASAL) in transgenic rice plants imparts resistance against multiple insect pests. *Scientific reports*, 8(1), 1-10.

Bommireddy, P. L., Leonard, B. R., Temple, J., Price, P., Emfinger, K., Cook, D., & Hardke, J. T. (2011). Field performance and seasonal efficacy profiles of transgenic cotton lines expressing Vip3A and VipCot against *Helicoverpa zea* (Boddie) and *Heliothis virescens* (F.).https://doi.org/10.18474/0749-8004-43.4.349.

Burke, W. G., Kaplanoglu, E., Kolotilin, I., Menassa, R., & Donly, C. (2019). RNA interference in the tobacco hornworm, *Manduca sexta*, using plastid-encoded long double-stranded RNA. *Frontiers in plant science*, 10, 313. https://doi.org/10.3389/fpls.2019.00313.

Caroline, N. M., Deogracious, P. M., George, M. T., James, R. M., Joel, W. D., & Paul, M. K. (2022). Identification of potential seed storage protein responsible for bruchid resistance in common bean landraces from Tanzania and Malawi. *African Journal of Biotechnology*, 21(1), 35-45.

Chakraborty, M., Reddy, P. S., Mustafa, G., Rajesh, G., Narasu, V. M., Udayasuriyan, V., & Rana, D. (2016). Transgenic rice expressing the cry2AX1 gene confers resistance to multiple lepidopteran pests. *Transgenic research*, 25(5), 665-678.<https://doi.org/10.1007/s11248-016-9954-4>.

Chang, X., Lu, Z., Shen, Z., Peng, Y., & Ye, G. (2017). Bitrophic and tritrophic effects of transgenic cry1Ab/cry2Aj maize on the beneficial, non target *Harmonia axyridis* (Coleoptera: Coccinellidae). *Environmental Entomology*, 46(5), 1171-1176. https://doi.org/10.1093/ee/nvx113.

Chen, B., Hu, J., Almeida, R., Liu, H., Balakrishnan, S., Covill-Cooke, C., ... & Huang, B. (2016). Expanding the CRISPR imaging toolset with *Staphylococcus aureus* Cas9 for simultaneous imaging of multiple genomic loci. *Nucleic acids research*, 44(8), e75-e75. https://doi.org/10.1093/nar/gkv1533.

Chen, W., Liu, C., Lu, G., Cheng, H., Shen, Z., & Wu, K. (2018). Effects of Vip3AcAa+ Cry1Ac cotton on midgut tissue in *Helicoverpa armigera* (Lepidoptera: Noctuidae). *Journal of Insect Science*, 18(4), 13. <https://doi.org/10.1093/jisesa/iey075>.

Coelho, J. S., Santos, N. D., Napoleão, T. H., Gomes, F. S., Ferreira, R. S., Zingali, R. B., ... & Paiva, P. M. (2009). Effect of *Moringa oleifera* lectin on development and mortality of *Aedes aegypti* larvae. *Chemosphere*, 77(7), 934-938. <https://doi.org/10.1016/j.chemosphere.2009.08.022>.

Das, A., Ghosh, P., & Das, S. (2018). Expression of *Colocasia esculenta* tuber agglutinin in Indian mustard provides resistance against *Lipaphis erysimi* and the expressed protein is non-allergenic. *Plant cell reports*, 37(6), 849-863.

de Albuquerque, L. P., de Sá Santana, G. M., Pontual, E. V., Napoleão, T. H., Coelho, L. C. B. B., & Paiva, P. M. G. (2012). Effect of *Microgramma vaccinifolia* rhizome lectin on survival and digestive enzymes of *Nasutitermes corniger* (Isoptera, Termitidae). *International biodeterioration & biodegradation*, 75, 158-166.

de Albuquerque, L. P., Procópio, T. F., da Silva Guedes, C. C., Pontual, E. V., Paiva, P. M. G., & Napoleão, T. H. (2020). Antinutritional effects of the chitin-binding lectin from *Microgramma vacciniifolia* rhizome (MvRL) on *Sitophilus zeamais*. *Journal of Stored Products Research*, 88, 101652.https://doi.org/10.1016/j.jspr.2020.101652.

de Oliveira, A. P. S., Agra-Neto, A. C., Pontual, E. V., de Albuquerque Lima, T., Cruz, K. C. V., de Melo, K. R., ... & Paiva, P. M. G. (2020). Evaluation of the insecticidal activity of *Moringa oleifera* seed extract and lectin (WSMoL) against *Sitophilus zeamais*. *Journal of stored products research*, 87, 101615.https://doi.org/10.1016/j.jspr.2020.101615.

de Santana Souza, C., Procópio, T. F., do Rego Belmonte, B., Paiva, P. M. G., de Albuquerque, L. P., Pontual, E. V., & Napoleão, T. H. (2018). Effects of *Opuntia ficus indica* lectin on feeding, survival, and gut enzymes of maize weevil, *Sitophilus zeamais*. *Applied Biological Chemistry*, 61(3), 337-343.

Di Lelio, I., Barra, E., Coppola, M., Corrado, G., Rao, R., & Caccia, S. (2022). Transgenic plants expressing immunosuppressive dsRNA improve entomopathogen efficacy against *Spodoptera littoralis* larvae. *Journal of Pest Science*, 1-16. <https://doi.org/10.1007/s10340-021-01467-z>.

Dong, Y., Yang, Y., Wang, Z., Wu, M., Fu, J., Guo, J., ... & Zhang, J. (2020). Inaccessibility to double‐stranded RNAs in plastids restricts RNA interference in *Bemisia tabaci* (whitefly). *Pest Management Science*, 76(9), 3168-3176. <https://doi.org/10.1002/ps.5871>

Douris, V., Steinbach, D., Panteleri, R., Livadaras, I., Pickett, J. A., Van Leeuwen, T., ... & Vontas, J. (2016). Resistance mutation conserved between insects and mites unravels the benzoylurea insecticide mode of action on chitin biosynthesis. *Proceedings of the National Academy of Sciences*, 113(51), 14692-14697.doi: 10.1073/pnas.1618258113.

Down, R. E., Fitches, E. C., Wiles, D. P., Corti, P., Bell, H. A., Gatehouse, J. A., & Edwards, J. P. (2006). Insecticidal spider venom toxin fused to snowdrop lectin is toxic to the peach‐potato aphid, *Myzus persicae* (Hemiptera: Aphididae) and the rice brown planthopper, *Nilaparvata lugens* (Hemiptera: Delphacidae). *Pest Management Science: formerly Pesticide Science*, 62(1), 77-85.https://doi.org/10.1002/ps.1119.

Duan, X., Hou, Q., Liu, G., Pang, X., Niu, Z., Wang, X., ... & Liang, R. (2018). Expression of *Pinellia pedatisecta* lectin gene in transgenic wheat enhances resistance to wheat aphids. *Molecules*, 23(4), 748.<https://doi.org/10.3390/molecules23040748>.

Fitches, E., Wilkinson, H., Bell, H., Bown, D. P., Gatehouse, J. A., & Edwards, J. P. (2004). Cloning, expression and functional characterisation of chitinase from larvae of tomato moth (*Lacanobia oleracea*): a demonstration of the insecticidal activity of insect chitinase. *Insect Biochemistry and Molecular Biology*, 34(10), 1037-1050. DOI: [10.1016/j.ibmb.2004.06.012](https://doi.org/10.1016/j.ibmb.2004.06.012).

Fujimoto, H., Itoh, K., Yamamoto, M., Kyozuka, J., & Shimamoto, K. O. (1993). Insect resistant rice generated by introduction of a modified δ-endotoxin gene of *Bacillus thuringiensis*. *Bio/technology*, 11(10), 1151-1155. DOI: [10.1038/nbt1093-1151](https://doi.org/10.1038/nbt1093-1151).

Ghareyazie, B., Alinia, F., Menguito, C. A., Rubia, L. G., de Palma, J. M., Liwanag, E. A., ... & Bennett, J. (1997). Enhanced resistance to two stem borers in an aromatic rice containing a synthetic cryIA (b) gene. *Molecular Breeding*, 3(5), 401-414. <https://doi.org/10.1023/a:1009695324100>.

Ghosh, G., Ganguly, S., Purohit, A., Chaudhuri, R. K., Das, S., & Chakraborti, D. (2017). Transgenic pigeonpea events expressing Cry1Ac and Cry2Aa exhibit resistance to *Helicoverpa armigera*. *Plant Cell Reports*, 36(7), 1037-1051. <https://doi.org/10.1007/s00299-017-2133-0>.

Gilles, A. F., Schinko, J. B., & Averof, M. (2015). Efficient CRISPR-mediated gene targeting and transgene replacement in the beetle *Tribolium castaneum*. *Development*, 142(16), 2832-2839.DOI: [10.1242/dev.125054](https://doi.org/10.1242/dev.125054).

Gowda, A., Rydel, T. J., Wollacott, A. M., Brown, R. S., Akbar, W., Clark, T. L., ... & Baum, J. A. (2016). A transgenic approach for controlling Lygus in cotton (vol 7, 12213, 2016). Nat. Commun., 11(1). DOI: [10.1038/s41467-020-14789-w](https://doi.org/10.1038/s41467-020-14789-w).

Grazziotin, M. A., Cabral, G. B., Ibrahim, A. B., Machado, R. B., & Aragao, F. J. (2020). Expression of the Arcelin 1 gene from *Phaseolus vulgaris* L. in cowpea seeds (*Vigna unguiculata* L.) confers bruchid resistance. *Annals of Applied Biology*, 176(3), 268-274. <https://doi.org/10.1111/aab.12568>.

Gunasekara, J. M. A., Jayasekera, G. A. U., Perera, K. L. N. S., & Wickramasuriya, A. M. (2017). Development of a Sri Lankan rice variety Bg 94-1 harbouring Cry2A gene of *Bacillus thuringiensis* resistant to rice leaf folder [*Cnaphalocrocis medinalis* (Guenée)]. *Journal of the National Science Foundation of Sri Lanka*, 45(2). https://doi.org/10.4038/jnsfsr.v45i2.8180.

Guo, Z., Li, Y., & Ding, S. W. (2019). Small RNA-based antimicrobial immunity. *Nature Reviews Immunology*, 19(1), 31-44. https://doi.org/10. 1038/s41577-018-0071-x.

Hilda, K., Bhuvaragavan, S., Kamatchi, R., Meenakumari, M., & Janarthanan, S. (2022). Cloning, expression and characterization of arcelin and its impact on digestive enzymes of the stored product insect pest, *Callosobruchus maculatus* (F.). *Pesticide Biochemistry and Physiology*, 180, 104982. DOI: [10.1016/j.pestbp.2021.104982](https://doi.org/10.1016/j.pestbp.2021.104982).

Huang, Y., Chen, Y., Zeng, B., Wang, Y., James, A. A., Gurr, G. M., ... & You, M. (2016). CRISPR/Cas9 mediated knockout of the abdominal-A homeotic gene in the global pest, diamondback moth (*Plutella xylostella*). *Insect Biochemistry and Molecular Biology*, 75, 98-106. DOI: [10.1016/j.ibmb.2016.06.004](https://doi.org/10.1016/j.ibmb.2016.06.004).

Hussain, T., Aksoy, E., Çalışkan, M. E., & Bakhsh, A. (2019). Transgenic potato lines expressing hairpin RNAi construct of molting-associated EcR gene exhibit enhanced resistance against Colorado potato beetle (*Leptinotarsa decemlineata*, Say). *Transgenic Research*, 28(1), 151-164.DOI: [10.1007/s11248-018-0109-7](https://doi.org/10.1007/s11248-018-0109-7).

Jadhav, M. S., Rathnasamy, S. A., Natarajan, B., Duraialagaraja, S., & Varatharajalu, U. (2020). Study of expression of indigenous *Bt* cry2AX1 gene in T_3_ progeny of cotton and its efficacy against *Helicoverpa armigera* (Hubner). *Brazilian Archives of Biology and Technology*, 63.

Jin, L., Wang, J., Guan, F., Zhang, J., Yu, S., Liu, S., ... & Wu, Y. (2018). Dominant point mutation in a tetraspanin gene associated with field-evolved resistance of cotton bollworm to transgenic *Bt* cotton. *Proceedings of the National Academy of Sciences*, 115(46), 11760-11765.

Katta, S., Talakayala, A., Reddy, M. K., Addepally, U., & Garladinne, M. (2020). Development of transgenic cotton (Narasimha) using triple gene Cry2Ab-Cry1F-Cry1Ac construct conferring resistance to lepidopteran pest. *Journal of biosciences*, 45(1), 1-11.

Khajuria, C., Vélez, A. M., Rangasamy, M., Wang, H., Fishilevich, E., Frey, M. L., ... & Siegfried, B. D. (2015). Parental RNA interference of genes involved in embryonic development of the western corn rootworm, *Diabrotica virgifera virgifera* LeConte. *Insect biochemistry and molecular biology*, 63, 54-62.DOI: [10.1016/j.ibmb.2015.05.011](https://doi.org/10.1016/j.ibmb.2015.05.011).

Khan, G. A., Bakhsh, A., Riazuddin, S., & Husnain, T. (2011). Introduction of cry1Ab gene into cotton (*Gossypium hirsutum*) enhances resistance against Lepidopteran pest (*Helicoverpa armigera*). *Spanish Journal of Agricultural Research*, 9(1), 296-302. http://dx.doi.org/10.5424/sjar/20110901-136-10.

Khoobdel, M., Rahimi, V., Ebadollahi, A., & Krutmuang, P. (2022). Evaluation of the potential of a lectin extracted from *Polygonum persicaria* L. as a biorational agent against *Sitophilus oryzae* L. *Molecules*, 27(3), 793. <https://doi.org/10.3390/molecules27030793>.

Kim, S. Y., Bengtsson, T., Olsson, N., Hot, V., Zhu, L. H., & Åhman, I. (2020). Mutations in two aphid-regulated β-1, 3-glucanase genes by CRISPR/Cas9 do not increase barley resistance to *Rhopalosiphum padi* L. *Frontiers in plant science*, 1043. <https://doi.org/10.3389/fpls.2020.01043>.

Koerniati, S., Sukmadjaja, D., & Samudra, I. M. (2020). C synthetic gene of CryIAb-CryIAc fusion to generate resistant sugarcane to shoot or stem borer. In *IOP Conference Series: Earth and Environmental Science* (Vol. 418, No. 1, p. 012069). IOP Publishing. doi:10.1088/1755-1315/418/1/012069.

Kola, V. S. R., Pichili, R., Padmakumari, A. P., Mangrauthia, S. K., Balachandran, S. M., & Madhav, M. S. (2019). Knockdown of acetylcholinesterase (AChE) gene in rice yellow stem borer, *Scirpophaga incertulas* (Walker) through RNA interference. *Agri Gene*, 11, 100081.

Koutroumpa, F. A., Monsempes, C., François, M. C., de Cian, A., Royer, C., Concordet, J. P., & Jacquin-Joly, E. (2016). Heritable genome editing with CRISPR/Cas9 induces anosmia in a crop pest moth. *Scientific Reports*, 6(1), 1-9. DOI: [10.1038/srep29620](https://doi.org/10.1038/srep29620).

Li, H., Guan, R., Guo, H., & Miao, X. (2015). New insights into an RNAi approach for plant defence against piercing‐sucking and stem‐borer insect pests. *Plant, cell & environment*, 38(11), 2277-2285.DOI: [10.1111/pce.12546](https://doi.org/10.1111/pce.12546).

Li, Y., Zhang, J., Chen, D., Yang, P., Jiang, F., Wang, X., & Kang, L. (2016). CRISPR/Cas9 in locusts: successful establishment of an olfactory deficiency line by targeting the mutagenesis of an odorant receptor co-receptor (Orco). *Insect biochemistry and molecular biology*, 79, 27-35.

Mamta, Reddy, K. R. K., & Rajam, M. V. (2016). Targeting chitinase gene of *Helicoverpa armigera* by host-induced RNA interference confers insect resistance in tobacco and tomato. *Plant Molecular Biology*, 90(3), 281-292. doi: 10.1007/s11103-015-0414-y. Epub 2015 Dec 10.

Maqbool, S. B., Riazuddin, S., Loc, N. T., Gatehouse, A. M., Gatehouse, J. A., & Christou, P. (2001). Expression of multiple insecticidal genes confers broad resistance against a range of different rice pests. *Molecular Breeding*, 7(1), 85-93.

Meade, T., Narva, K., Storer, N. P., Sheets, J. J., Burton, S. L. and Woosley, A. T. (2017). *U.S. Patent No. 9,556,453*. Washington, DC: U.S. Patent and Trademark Office.

Meccariello, A., Monti, S. M., Romanelli, A., Colonna, R., Primo, P., Inghilterra, M. G.,…..& Saccone, G. (2017). Highly efficient DNA-free gene disruption in the agricultural pest *Ceratitis capitata* by CRISPR-Cas9 ribonucleoprotein complexes. *Scientific Reports*, 7, 1-11.

Meng, F., Li, Y., Zang, Z., Li, N., Ran, R., Cao, Y., ... & Li, W. (2017). Expression of the double‐stranded RNA of the soybean pod borer *Leguminivora glycinivorella* (Lepidoptera: Tortricidae) ribosomal protein Po gene enhances the resistance of transgenic soybean plants. *Pest management science*, 73(12), 2447-2455.

Mi, X., Liu, X., Yan, H., Liang, L., Zhou, X., Yang, J., ... & Zhang, N. (2017). Expression of the *Galanthus nivalis agglutinin* (GNA) gene in transgenic potato plants confers resistance to aphids. *Comptes rendus biologies*, 340(1), 7-12. doi: 10.1016/j.crvi.2016.

Muddanuru, T., Polumetla, A. K., Maddukuri, L., & Mulpuri, S. (2019). Development and evaluation of transgenic castor (*Ricinus communis* L.) expressing the insecticidal protein Cry1Aa of *Bacillus thuringiensis* against lepidopteran insect pests. *Crop Protection*, 119, 113-125. https://doi.org/10.1016/j.cropro.2019.01.016.

Nakasu, E. Y., Edwards, M. G., Fitches, E., Gatehouse, J. A., & Gatehouse, A. M. (2014). Transgenic plants expressing ω-ACTX-Hv1a and snowdrop lectin (GNA) fusion protein show enhanced resistance to aphids. *Frontiers in plant science*, 5, 673. <https://doi.org/10.3389/fpls.2014.00673>.

Napoleão, T. H., do Rego Belmonte, B., Pontual, E. V., de Albuquerque, L. P., Sá, R. A., Paiva, L. M., ... & Paiva, P. M. G. (2013). Deleterious effects of *Myracrodruon urundeuva* leaf extract and lectin on the maize weevil, *Sitophilus zeamais* (Coleoptera, Curculionidae). *Journal of stored products research*, 54, 26-33. **DOI :** [10.1016/j.jspr.2013.04.002](http://dx.doi.org/10.1016/j.jspr.2013.04.002).

Nayak, P., Basu, D., Das, S., Basu, A., Ghosh, D., Ramakrishnan, N. A., ... & Sen, S. K. (1997). Transgenic elite indica rice plants expressing CryIAc∂-endotoxin of *Bacillus thuringiensis* are resistant against yellow stem borer (*Scirpophaga incertulas*). *Proceedings of the National Academy of Sciences*, 94(6), 2111-2116. doi: 10.1073/pnas.94.6.2111.

Ni, M., Ma, W., Wang, X., Gao, M., Dai, Y., Wei, X., ... & Zhu, Z. (2017). Next‐generation transgenic cotton: pyramiding RNAi and *Bt* counters insect resistance. *Plant biotechnology journal*, 15(9), 1204-1213. https://doi.org/10.1111/pbi.12709.

Niu, X., Kassa, A., Hu, X., Robeson, J., McMahon, M., Richtman, N. M., ... & Wu, G. (2017). Control of western corn rootworm (*Diabrotica virgifera virgifera*) reproduction through plant-mediated RNA interference. *Scientific reports*, 7(1), 1-13. <http://dx.doi.org/10.1038/s41598-017-12638-3>.

Qin, D., Liu, X. Y., Miceli, C., Zhang, Q., & Wang, P. W. (2019). Soybean plants expressing the *Bacillus thuringiensis* cry8-like gene show resistance to *Holotrichia parallela*. *BMC biotechnology*, 19(1), 1-12. https://doi.org/10.1186/s12896-019-0563-1.

Qiu, L., Fan, J., Zhang, B., Liu, L., Wang, X., Lei, C., ... & Ma, W. (2017). RNA interference knockdown of aminopeptidase N genes decrease the susceptibility of *Chilo suppressalis* larvae to Cry1Ab/Cry1Ac and Cry1Ca-expressing transgenic rice. *Journal of invertebrate pathology*, 145, 9-12.

Qiu, L., Sun, Y., Jiang, Z., Yang, P., Liu, H., Zhou, H., ... & Ma, W. (2019). The midgut V‐ATPase subunit A gene is associated with toxicity to crystal 2Aa and crystal 1Ca‐expressing transgenic rice in *Chilo suppressalis*. *Insect molecular biology*, 28(4), 520-527. https://doi.org/10.1111/imb.12570.

Rajadurai, G., Kalaivani, A., Varanavasiyappan, S., Balakrishnan, N., Udayasuriyan, V., Sudhakar, D., & Natarajan, N. (2018). Generation of insect resistant marker-free transgenic rice with a novel cry2AX1 gene. *Electronic Journal of Plant Breeding*, 9(2), 723-732.

Rani, S., Sharma, V., Hada, A., Bhattacharya, R. C., & Koundal, K. R. (2017b). Fusion gene construct preparation with lectin and protease inhibitor genes against aphids and efficient genetic transformation of *Brassica juncea* using cotyledons explants. *Acta Physiologiae Plantarum*, 39(5), 1-13. DOI 10.1007/s11738-017-2415-8.

Riaz, S., Nasir, I. A., Bhatti, M. U., Adeyinka, O. S., Toufiq, N., Yousaf, I., & Tabassum, B. (2020). Resistance to *Chilo infuscatellus* (Lepidoptera: Pyraloidea) in transgenic lines of sugarcane expressing *Bacillus thuringiensis* derived Vip3A protein. *Molecular Biology Reports*, 47(4), 2649-2658. doi: 10.1007/s11033-020-05355-0.

Ribeiro, T. P., Arraes, F. B. M., Lourenço‐Tessutti, I. T., Silva, M. S., Lisei‐de‐Sá, M. E., Lucena, W. A., ... & Grossi‐de‐Sa, M. F. (2017). Transgenic cotton expressing Cry10Aa toxin confers high resistance to the cotton boll weevil. *Plant biotechnology journal*, 15(8), 997-1009.

Sakthi, A. R., Naveenkumar, A., Deepikha, P. S., Balakrishnan, N., Kumar, K. K., Devi, E. K., ... & Balasubramanian, P. (2015). Expression and inheritance of chimeric cry2AX1 gene in transgenic cotton plants generated through somatic embryogenesis. *In Vitro Cellular & Developmental Biology-Plant*, 51(4), 379-389.

Santos, N. D. D. L., de Moura, K. S., Napoleao, T. H., Santos, G. K. N., Coelho, L. C. B. B., Navarro, D. M. D. A. F., & Paiva, P. M. G. (2012). Oviposition-stimulant and ovicidal activities of *Moringa oleifera* lectin on *Aedes aegypti*. *Plos One*, 7(9), 1-8. https://doi.org/10.1371/journal.pone.0044840.

Santos, N. D. L., Napoleão, T. H., Benevides, C. A., Albuquerque, L. P., Pontual, E. V., Oliveira, A. P. S., ... & Paiva, P. M. G. (2020). Effect of gamma irradiation of *Moringa oleifera* seed lectin on its larvicidal, ovicidal, and oviposition-stimulant activities against *Aedes aegypti*. *South African Journal of Botany*, 129, 3-8.

Sawardekar, S. V., Katageri, I. S., Salimath, P. M., Kumar, P. A., & Kelkar, V. G. (2017). Standardization of in-vitro genetic transformation technique in chickpea (*Cicer arietinum* L.) for pod-borer resistance. *Advanced Agricultural Research and Technology*, 1(2).

Selale, H., Dağlı, F., Mutlu, N., Doğanlar, S., & Frary, A. (2017). Cry1Ac-mediated resistance to tomato leaf miner (*Tuta absoluta*) in tomato. *Plant Cell, Tissue and Organ Culture (PCTOC)*, 131(1), 65-73.

Sétamou, M., Bernal, J. S., Legaspi, J. C., Mirkov, T. E., & Legaspi Jr, B. C. (2002). Evaluation of lectin-expressing transgenic sugarcane against stalk borers (Lepidoptera: Pyralidae): effects on life history parameters. *Journal of Economic Entomology*, 95(2), 469-477.<https://doi.org/10.1603/0022-0493-95.2.469>.

Siddiqui, H. A., Asif, M., Asad, S., Naqvi, R. Z., Ajaz, S., Umer, N., ... & Mansoor, S. (2019). Development and evaluation of double gene transgenic cotton lines expressing Cry toxins for protection against chewing insect pests. *Scientific reports*, 9(1), 1-7. doi: 10.1038/s41598-019-48188-z.

Singh, S., Kumar, N. R., Maniraj, R., Lakshmikanth, R., Rao, K. Y. S., Muralimohan, N., ... & Sreevathsa, R. (2018). Expression of Cry2Aa, a *Bacillus thuringiensis* insecticidal protein in transgenic pigeon pea confers resistance to gram pod borer, *Helicoverpa armigera*. *Scientific reports*, 8(1), 1-12. DOI:10.1038/s41598-018-26358-9.

Soliman, H. I., Abo-El-Hasan, F. M., El-Seedy, A. S., & Mabrouk, Y. M. (2021). Agrobacterium-mediated transformation of tomato (*Lycopersicon esculentum* mill.) using a synthetic cry1ab gene for enhanced resistance against *Tuta absoluta* (Meyrick). *Journal of Microbiology, Biotechnology and Food Sciences*, 67-74. <https://doi.org/10.15414/jmbfs.2017.7.1.67-74>.

Stoger, E., Williams, S., Christou, P., Down, R. E., & Gatehouse, J. A. (1999). Expression of the insecticidal lectin from snowdrop (*Galanthus nivalis agglutinin*; GNA) in transgenic wheat plants: effects on predation by the grain aphid *Sitobion avenae*. *Molecular Breeding*, 5(1), 65-73. **DOI:**10.1023/a:1009616413886.

Sudhakar, D., Fu, X., Stoger, E., Williams, S., Spence, J., Brown, D. P., ... & Christou, P. (1998). Expression and immunolocalisation of the snowdrop lectin, GNA in transgenic rice plants. *Transgenic Research*, 7(5), 371-378. doi: 10.1023/a:1008856703464.

Trung, N. P., Fitches, E., & Gatehouse, J. A. (2006). A fusion protein containing a lepidopteran-specific toxin from the South Indian red scorpion (*Mesobuthus tamulus*) and snowdrop lectin shows oral toxicity to target insects. *BMC biotechnology*, 6(1), 1-12. https://doi.org/10.1186/ 1472-6750-6-18. doi:10.1186/1472-6750-6-18.

Tu, J., Zhang, G., Datta, K., Xu, C., He, Y., Zhang, Q., ... & Datta, S. K. (2000). Field performance of transgenic elite commercial hybrid rice expressing *Bacillus thuringiensis* δ-endotoxin. *Nature biotechnology*, 18(10), 1101-1104. doi: 10.1038/80310.

Vanti, G. L., Katageri, I. S., Inamdar, S. R., Hiremathada, V., & Swamy, B. M. (2018). Potent insect gut binding lectin from *Sclerotium rolfsii* impart resistance to sucking and chewing type insects in cotton. *Journal of Biotechnology*, 278, 20-27. doi: 10.1016/j.jbiotec.2018.04.018.

Wang, J., Ma, H., Zuo, Y., Yang, Y., & Wu, Y. (2020a). CRISPR‐mediated gene knockout reveals nicotinic acetylcholine receptor (nAChR) subunit α6 as a target of spinosyns in *Helicoverpa armigera*. *Pest Management Science*, 76(9), 2925-2931. doi: 10.1002/ps.5889.

Wang, J., Zhang, H., Wang, H., Zhao, S., Zuo, Y., Yang, Y., & Wu, Y. (2016). Functional validation of cadherin as a receptor of *Bt* toxin Cry1Ac in *Helicoverpa armigera* utilizing the CRISPR/Cas9 system. *Insect biochemistry and molecular biology*, 76, 11-17. doi: 10.1016/j.ibmb.2016.06.008.

Wang, X., Xu, Y., Huang, J., Jin, W., Yang, Y., & Wu, Y. (2020b). CRISPR-mediated knockout of the ABCC2 gene in *Ostrinia furnacalis* confers high-level resistance to the *Bacillus thuringiensis* Cry1Fa toxin. *Toxins*, 12(4), 246. <https://dx.doi.org/10.3390%2Ftoxins12040246>.

Wünn, J., Klöti, A., Burkhardt, P. K., Biswas, G. C. G., Launis, K., Iglesias, V. A., & Potrykus, I. (1996). Transgenic indica rice breeding line IR58 expressing a synthetic crylA (b) gene from *Bacillus thuringiensis* provides effective insect pest control. *Bio/technology*, 14(2), 171-176.

Xia, J., Guo, Z., Yang, Z., Han, H., Wang, S., Xu, H., ... & Zhang, Y. (2021). Whitefly hijacks a plant detoxification gene that neutralizes plant toxins. *Cell*, 184(7), 1693-1705. doi: 10.1016/j.cell.2021.02.014.

Xu, C., Cheng, J., Lin, H., Lin, C., Gao, J. & Shen, Z. (2018). Characterization of transgenic rice expressing fusion protein Cry1Ab/Vip3A for insect resistance. *Scientific Reports,* 8, 1-8. DOI:10.1038/s41598-018-34104-4.

Yan, S., Qian, J., Cai, C., Ma, Z., Li, J., Yin, M., ... & Shen, J. (2020). Spray method application of transdermal dsRNA delivery system for efficient gene silencing and pest control on soybean aphid *Aphis glycines*. *Journal of Pest Science*, 93(1), 449-459.

Zhang, J., Khan, S. A., Hasse, C., Ruf, S., Heckel, D. G., & Bock, R. (2015). Full crop protection from an insect pest by expression of long double-stranded RNAs in plastids. *Science*, 347(6225), 991-994.doi: 10.1126/science.1261680.

Zhong, Y., Ahmed, S., Deng, G., Fan, W., Zhang, P., & Wang, H. (2019). Improved insect resistance against *Spodoptera litura* in transgenic sweet potato by over expressing Cry1Aa toxin. *Plant Cell Reports*, 38(11), 1439-1448. doi: 10.1007/s00299-019-02460-8.

Zuo, Y., Wang, H., Xu, Y., Huang, J., Wu, S., Wu, Y., & Yang, Y. (2017). CRISPR/Cas9 mediated G4946E substitution in the ryanodine receptor of *Spodoptera exigua* confers high levels of resistance to diamide insecticides. *Insect Biochemistry and Molecular Biology*, 89, 79-85. doi: 10.1016/j.ibmb.2017.09.005.

Zuo, Y., Xue, Y., Lu, W., Ma, H., Chen, M., Wu, Y., ... & Hu, Z. (2020). Functional validation of nicotinic acetylcholine receptor (nAChR) α6 as a target of spinosyns in *Spodoptera exigua* utilizing the CRISPR/Cas9 system. *Pest Management Science*, 76(7), 2415-2422.
